# Machine-Learning for Phenotyping and Prognostication of Myocardial Infarction and Injury in Suspected Acute Coronary Syndrome

**DOI:** 10.1016/j.jacadv.2024.101011

**Published:** 2024-06-19

**Authors:** Ehsan Khan, Kristina Lambrakis, Zhibin Liao, Joey Gerlach, Tom Briffa, Louise Cullen, Adam J. Nelson, Johan Verjans, Derek P. Chew

**Affiliations:** a College of Medicine & Public Health, Flinders University of South Australia, Adelaide, Australia; b Department of Cardiology, Southern Adelaide Local Health Network, Adelaide, Australia; c Australian Institute of Machine Learning, University of Adelaide, Adelaide, Australia; d School of Population and Global Health, University of Western Australia, Perth, Australia; e Emergency and Trauma Centre, Royal Brisbane and Women’s Hospital, Brisbane, Australia; f School of Public Health, Queensland University of Technology, Brisbane, Australia; g School of Medicine, University of Queensland, Brisbane, Australia; h Department of Cardiology, Victorian Heart Hospital, Melbourne, Australia; i Heart and Vascular Health, South Australian Health and Medical Research Institute, Adelaide, Australia; j Monash Victorian Heart Institute, Monash University, Melbourne, Australia

**Keywords:** artificial intelligence, machine learning, myocardial infarction, myocardial injury, troponin

## Abstract

**Background:**

Clinical work-up for suspected acute coronary syndrome (ACS) is resource intensive.

**Objectives:**

This study aimed to develop a machine learning model for digitally phenotyping myocardial injury and infarction and predict 30-day events in suspected ACS patients.

**Methods:**

Training and testing data sets, predominantly derived from electronic health records, included suspected ACS patients presenting to 6 and 26 South Australian hospitals, respectively. All index presentations and 30-day death and myocardial infarction (MI) were adjudicated using the Fourth Universal Definition of MI. We developed 2 diagnostic prediction models which phenotype myocardial injury and infarction according to the Fourth UDMI (chronic myocardial injury vs acute myocardial injury patterns, the latter further differentiated into acute non-ischaemic myocardial injury, Types 1 and 2 MI) using eXtreme Gradient Boosting (XGB) and deep-learning (DL). We also developed an event prediction model for risk prediction of 30-day death or MI using XGB. Analyses were performed in Python 3.6.

**Results:**

The training and testing data sets had 6,722 and 8,869 participants, respectively. The diagnostic prediction XGB and deep learning models achieved an area under the curve of 99.2% ± 0.1% and 98.8% ± 0.2%, respectively, for differentiating an acute myocardial injury *pattern* from no injury or chronic myocardial injury *pattern* and achieved 95.5% ± 0.2% and 94.6% ± 0.9%, respectively, for differentiating type 1 MI from type 2 MI or acute nonischemic myocardial injury. The 30-day death/MI event prediction model achieved an area under the curve of 88.5% ± 0.5%.

**Conclusions:**

Machine learning models can digitally phenotype suspected ACS patients at index presentation and predict subsequent events within 30 days. These models require external validation in a randomized clinical trial to evaluate their impact in clinical practice.

Assessment of patients with suspected acute coronary syndrome (ACS) in the emergency department (ED) is often protracted and resource intensive accounting for health system costs of over $1 billion/annum across Australia and over $3 billion/annum in the United States.^1^^,^^2^ While ED clinical work-up is necessary to exclude acute, life-threatening conditions, it is critical to recognize that up to 85% of these patients are not ultimately diagnosed with an ACS.

Widespread clinical adoption of high-sensitivity cardiac troponin (hs-cTn) assays has not necessarily made clinical decision-making easier with a substantial proportion of troponin elevations resulting from myocardial injury-related diagnoses other than type 1 myocardial infarction (T1MI).^3^^,^^4^ Patients with non-T1MI, including type 2-MI (T2MI), acute and chronic myocardial injury, previously may have been dismissed as having “troponinemia” (ie, elevated troponin considered to be benign), however prior evidence demonstrates non-T1MI diagnostic classifications to have a high risk of poor clinical outcomes at 1-year demanding a paradigm shift in clinical decision-making.^3^^,^^5^, ^6^, ^7^ Correct diagnostic phenotyping in clinical practice according to the latest iteration of the Universal Definition of MI (UDMI) is imperative to allow early differentiation of potential T1MI patients for timely administration of well-established evidence-based ACS pharmacotherapies and consideration of early invasive management. Furthermore, accurate delineation of non-T1MI cohorts will enable the establishment and maturation of an evidence-base aimed to improve clinical outcomes where practice currently remains evidence-free and largely consensus-based.^8^, ^9^, ^10^, ^11^

Artificial intelligence (AI) represents a unique opportunity to support diagnosis and risk stratification in real-time clinical practice. Among the abundance of suspected ACS patients who present to EDs, utilizing an AI model for digital phenotyping of MI and myocardial injury could facilitate diagnosis and provide near-term risk assessment more accurately and consistently, thereby enabling improved clinical decision-making and robust evidence synthesis. The availability of electronic health record (EHR) data is ever-increasing in clinical practice and the capability of AI to process large data is hence advantageous in this clinical environment. The implementation of AI in clinical practice using real-time EHR data for clinical decision support may represent the next step in harnessing the benefits of EHR. Our aim was to develop machine learning (ML) models with a supervised learning approach with clinically acceptable capability to diagnose types of MI and myocardial injury at ED presentation and predict near-term events among suspected ACS patients.

## Methods

### Setting and study participants

Data from patients presenting to the ED of 7 metropolitan and 19 regional hospitals across South Australia with suspected ACS and consequent measurement of high-sensitivity cardiac troponin T (hs-cTnT) concentrations were utilized. The methods section (including the Supplemental Methods) is reported according to the TRIPOD statement and the recommendations made by Stevens et al.^12^^,^^13^

A total of 3 data sets were available for analysis. All cohort studies were conducted in accordance with the Declaration of Helsinki and approved by the local human research ethics committee. The data sets are outlined in the Supplemental Methods.

### Pathology and clinical data

All sites involved used the same pathology service hence we deterministically linked all available pathology data using patient identifiers. This pathology service deployed the same hs-cTnT assay at all sites (Roche Diagnostics Cobas Elecsys Gen 5 tested in plasma, limit of detection [LoD]: 5 ng/L, limit of quantification: 6 ng/L, 10% CV: 13 ng/L, 99th percentile: 14 ng/L) since June 2011. Importantly, hs-cTnT concentrations to the LoD were available for all participants in the study. Pathology data including troponin concentrations measured only *within the first 24 hours of index presentation* were used for analysis.

Patient comorbidities and demographic data were collected during the study for randomized controlled trial data or were coded from administrative records for EHR data from primary and secondary diagnoses using the International Classification of Diseases, version-10 Australian Modified code from prior presentations over at least 5 years. If patients were not observed in the hospital administrative records within the previous 5-year period, they were coded as not having the comorbidity.

### Assessment of clinical outcomes

All index presentations from all data sets were clinically adjudicated by the clinical events committees for the RCT data and by a qualified cardiologist for EHR data according to the Fourth UDMI into the following constituents: nonelevated hs-cTnT, chronic myocardial injury, acute nonischemic myocardial injury, T1MI, and T2MI.^14^ Adjudicators reviewed demographic and clinical data, including pathology, as well as coded diagnostic and procedural information for clinical adjudication of index presentations according to the Fourth UDMI (Supplemental Methods). Similarly, MI at 30 days was clinically adjudicated using the process above and death (all-cause mortality) was captured using linked data to the state Births, Deaths, and Marriages registry.

### Model development

1) Diagnostic prediction of index presentations: A supervised learning approach was taken using eXtreme Gradient Boosting (XGboost; XGB) and deep learning (DL) with multiple-layer perceptron to develop 2 separate models.

For the XGB model, the Fourth UDMI was used as a framework by building a 2-level model with a binary classifier. Level 1 was focussed on differentiating the *pattern* of hs-cTnT elevation: acute injury *pattern* vs chronic injury *pattern* or nonelevated hs-cTnT and level 2 on further differentiating acute injury *pattern* into T1MI vs T2MI or acute nonischemic myocardial injury (ie, as a composite). This approach reflects the systematic approach a clinician may take in applying the Fourth UDMI, as level 1 delineates patients potentially suitable for discharge (nonelevated cTn and chronic myocardial injury) and level 2 delineates and identifies patients where established evidence-based management strategies are to be initiated (ie, in T1MI).

The DL model was built using a 5-level classifier with nonelevated hs-cTnT, chronic myocardial injury, acute nonischemic myocardial injury, T1MI, and T2MI as individual constituents. The results were aggregated according to level 1 and 2 from the XGB model for comparison purposes.

2) Event prediction for short-term clinical outcomes: A supervised approach was taken for this model for prediction of death or MI at 30 days using XGB.

The data sets were first split into 3 subsets: training, validation, and testing. For XGB models, we used repeated and stratified 5-fold cross validation on a combined set (training and validation subsets) and for the DL model, due to a longer training time, we used the training and validation subsets separately. The performance of both models was reported on the same test subsets. To reduce the performance variation due to random data set splits and model initialization, we repeated testing up to a maximum of 50 submodels with different data set splits (Supplemental Figure 4). The mean of the test performance statistics with 95% confidence intervals were reported. In addition to the overall testing data set, we reported the model performance in the data set 1 and 2 subsets to assess the performance of the model when electrocardiogram (ECG) data (data set 2) were available and to ensure the model was not biased toward a particular data set. Data preprocessing and transformation, defining ML analysis architecture and ML methods, and the computational architecture are described in the Supplemental Methods.

### Exploring model performance with lower repetition for clinical deployment

Since our plan was to deploy these models in clinical practice, reducing the model processing time for real-time feedback at the front-end was vital. Hence, we explored models with the least number of repetitions required to achieve an area under the curve (AUC) within 1% of the corresponding 50-repetition model.

### Sensitivity analysis

The XGB and DL models were retrained using the following input data to determine the incremental gain in model performance with each step of additional data: 1) clinical data including age, gender, and prior comorbidity variables including prior hypertension, diabetes, smoking, ACS, acute MI, heart failure, cerebrovascular accident, chronic obstructive pulmonary disease, and chronic kidney disease only; 2) all available troponin data only; 3) the addition of all available troponin data to (1); 4) the addition of ECG data to (3); 5) the addition of renal function, hemoglobin, and white blood cell count to (4); and 6) all input data. We also assessed model performance in prespecified subgroups including age, gender, and time of troponin data from presentation.

### Performance measurement and target

We reported the following performance metrics for comparison and to determine the clinical utility for each of the models: sensitivity, specificity, positive predictive value (PPV), negative predictive value (NPV), and AUC. These performance metrics were reported for the detection (ie, true positive) of acute myocardial injury *pattern* at level 1 and for the detection of T1MI at level 2. Isotonic regression was used for model calibration and we reported the Brier score (reported as percentage), calibration slope and intercept, and mean calibration (“calibration-in-the-large”) for each model—for the diagnostic prediction models, probabilities were calibrated separately for level 1 and 2.^15^, ^16^, ^17^, ^18^ We determined prespecified performance goals to be met prior to clinical deployment. Our performance target for the diagnostic prediction model was to achieve a NPV of ≥99% for level 1 to limit the misclassification of acute injury patients (false negatives) to ≤1% given the clinical consensus of an acceptable miss rate for discharged patients presenting with suspected ACS within the ED.^19^ The goal for level 2 in diagnostic prediction and event prediction models was to maximize the NPV and PPV without a specific value.

### Statistical analysis

Baseline characteristics for continuous variables were reported as median (IQR) and as proportions for categorical variables. We reported the following test performance statistics for each of the models with confidence intervals within the overall testing data set and within the testing subsets of data set 1 and 2: sensitivity, specificity, NPV, and AUC. ML performance metrics such as precision, recall, F1, and accuracy were also available however were not reported as they did not reflect our clinical priorities. The learnt feature importance for these models was illustrated using relative values generated by XGB (normalized gain) and DL (SHAP values) models—only features with values >0.01 were reported given the large number of features included.^20^ In the sensitivity analyses, we reported the AUC for each of the models and at each level for the diagnostic prediction models.

## Results

### Baseline characteristics

**Figure 1:**
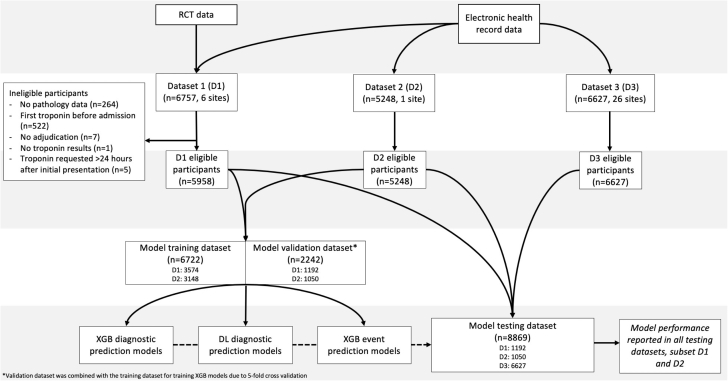
**Study Flow Diagram** DL = deep learning; XGB = eXtreme Gradient Boosting.

There were 6,757 participants in data set 1 of which 798 were ineligible resulting in 5,958 participants. Data sets 2 and 3 had 5,248 and 6,627 participants, respectively, with no ineligible participants (Figure 1). Participants in data set 3 were younger but had a lower proportion of females compared to data sets 1 and 2. Data set 2 participants had higher proportions of cardiovascular and non-cardiovascular comorbidities compared to participants in data sets 1 and 3. A median of 2 troponin concentrations were measured within 24 hours of presentation in data sets 1 and 3 compared to 3 measurements in data set 2. The time to first troponin concentration measurement varied between 37.5 and 51.0 mins across the 3 data sets (Table 1). The availability of all model features is reported in Supplemental Table 6. ECG data were only available in data set 2 where 56.4% and 16.4% of participants were classified to be in sinus rhythm and atrial fibrillation, respectively. Ischemic changes including T wave inversion, ST depression were found in 30.0% and 21.3% of participants, respectively (Supplemental Table 7).

**Table 1:** Baseline Characteristics

	Data Set 1 (n = 5,958)	Data Set 2 (n = 5,248)	Data Set 3 (n = 6,627)	Data Sets 1 and 2 (n = 11,206)	Overall (N = 17,833)
Age, y	63.1 (50.7-76.9)	72.4 (58.8-83.3)	60.7 (43.2-75.2)	67.6 (53.8-80.2)	65.2 (50.3-78.4)
Female, %	53.9	54.5	46.8	54.2	51.5
Prior comorbidities, %					
Hypertension	21.2	43.5	27.2	31.6	30.0
Diabetes	14.3	18.8	13.3	16.4	15.2
Smoking	17.1	34.6	27.6	25.3	26.1
AMI	10.2	6.9	4.6	8.7	7.2
Heart failure	8.7	13.2	7.0	10.8	9.4
CVA	3.7	5.5	4.1	4.6	4.4
COPD	6.6	20.0	15.5	12.9	13.9
CKD	10.7	10.6	5.3	10.7	8.7
eGFR	82.4 (64.2-98.6)	75 (52-90)	89 (67-90)	79.6 (58-90)	83 (61-90)
Troponin count ≤24 h	2 (2-3)	3 (3-3)	2 (2-3)	2 (2-3)	2 (1-2)
≤2 troponin concentrations measured, %	61.8	76.7	85.8	68.8	75.1
Time to first troponin, mins	37.5 (24.1-63.7)	46.0 (28.0-97.0)	51.0 (28.0-105.0)	41.0 (26.0-77.5)	44.0 (26.5-88.1)
Adjudicated outcomes, %					
T1MI	4.8	6.8	9.2	5.8	7.0
T2MI	3.7	5.1	10.2	4.4	6.5
Acute nonischemic myocardial injury	8.5	8.2	7.5	8.4	8.0
Chronic myocardial injury	21.8	43.0	6.4	31.8	22.4
Nonelevated troponin	61.3	36.8	66.7	49.8	56.1
Death/MI at 30 days	3.9	4.8	5.3	4.3	4.7

Values are median (IQR) or %.

AMI = acute myocardial infarction; CKD = chronic kidney disease; COPD = chronic obstructive pulmonary disease; CVA = cerebrovascular accident; eGFR = estimated glomerular filtration rate; MI = myocardial infarction; T1MI = type 1 myocardial infarction; T2MI = type 2 myocardial infarction.

### Clinical adjudication of index presentations

Acute nonischemic myocardial injury and death/MI at 30 days were similar between the 3 data sets. Data sets 1 and 2 had the highest rate of nonelevated troponin and chronic myocardial injury, respectively. Data set 3 had the highest rate of T1MI and T2MI (Table 1).

### Overall model performance

#### Diagnostic prediction

**Figure 2:**
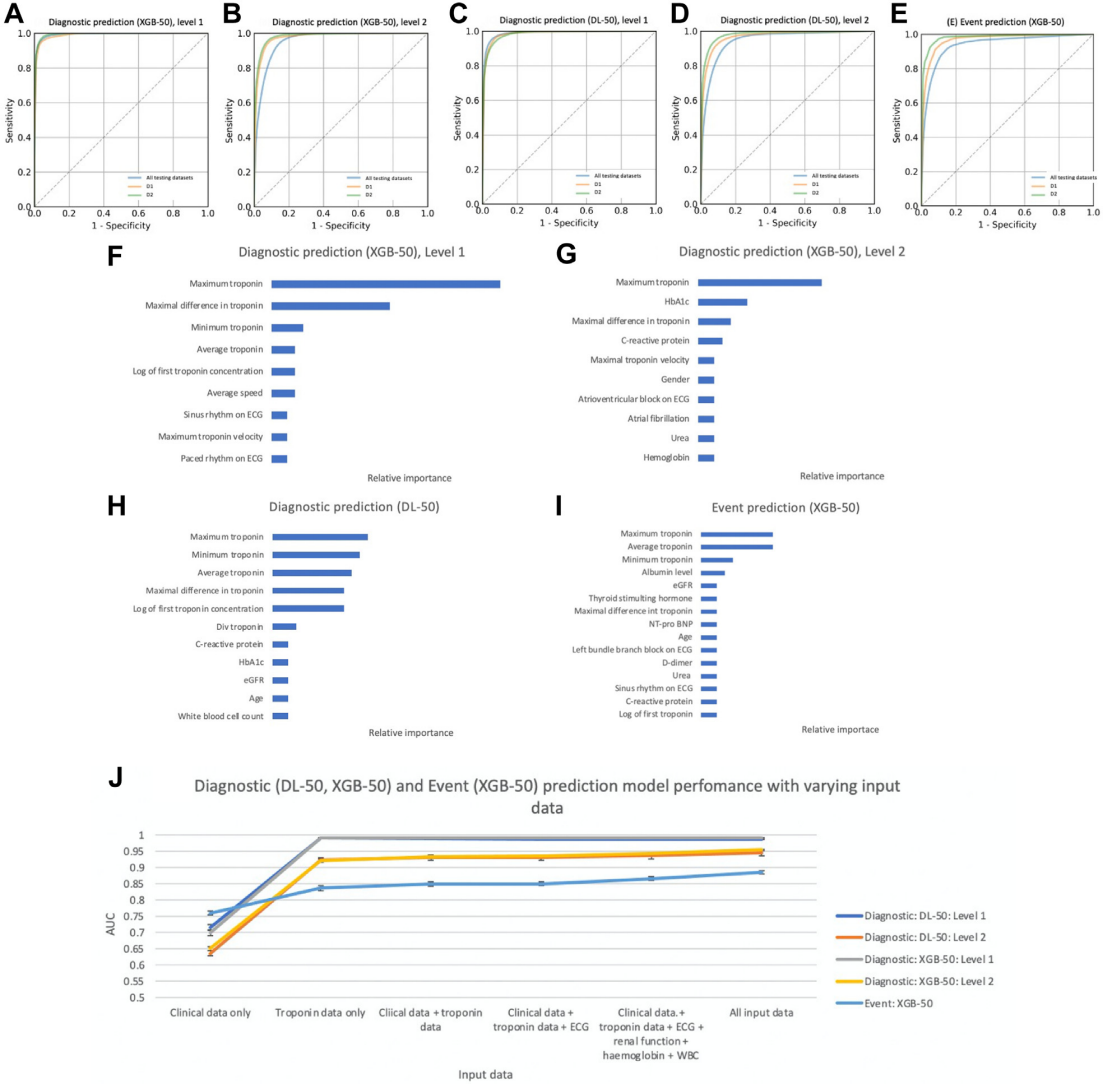
Model Performance and Feature Importance (A to E) ROC curves for diagnostic and event prediction models. (F to I) Feature importance. (J) Model performance according to input data. AUC = area under the curve; eGFR = estimated glomerular filtration rate; ROC = receiver operator characteristic; other abbreviations as in Figure 1.

The performance for the XGB-50 and DL-50 diagnostic prediction models in all training and testing data sets is summarized in the Central Illustration, Figure 2, and Supplemental Tables 1 and 4. XGB-50 and DL-50 had an AUC of 99.2% ± 0.1% and 98.8% ± 0.2% for level 1 and 95.5% ± 0.2% and 94.6% ± 0.9% for level 2, respectively, in the overall testing data set (Figures 2A to 2D). Both XGB-50 and DL-50 models optimized for true positive rate met the performance target for level 1 and 2. There were minor differences in performance within the subsets of data set 1 and 2 (Supplemental Tables 2 and 3). These models had low Brier scores at level 1 and 2 (XGB-50: L1 - 3.6% ± 0.2%, L2 - 4.6% ± 0.1%; DL: L1 - 3.9% ± 0.2%, L2 - 3.6% ± 0.2%) (Supplemental Tables 1 to 3).

**Central Illustration:**
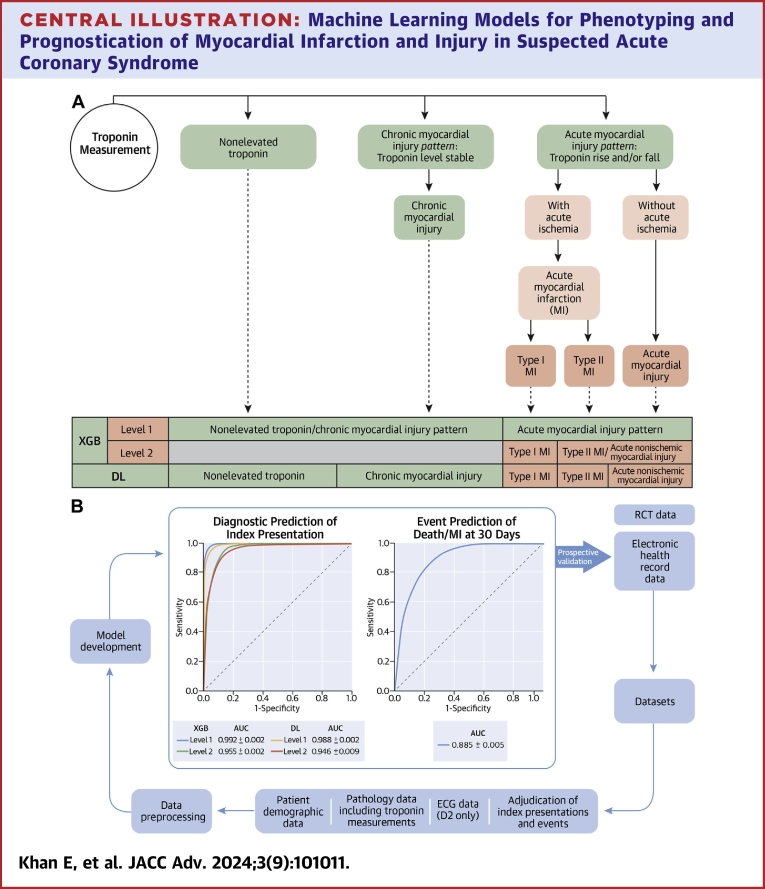
Machine Learning Models for Phenotyping and Prognostication of Myocardial Infarction and Injury in Suspected Acute Coronary Syndrome (A) Diagnostic classification according to fourth universal definition of MI (Thygesen et al^14^) and associated diagnostic prediction for XGB and DL models. (B) Summary of model development and performance. MI = myocardial infarction; other abbreviations as in Figures 1 and 2.

Feature importance using normalized gain and SHAP values for the XGB-50 and DL-50 models, respectively, are summarized in Figures 2F to 2H. The features of importance in XGB-50 for level 1 included derivatives of the troponin profile. For level 2, maximum troponin remains the most important feature although clinical differentiators also have an influence including biochemical results (hemoglobin A1c [HbA1c], C-reactive protein [CRP], hemoglobin, and urea), gender and presence of atrial fibrillation or atrioventricular block on ECG.

Feature importance for DL-50 models was similar as the troponin profile has a large influence on the models with the first 5 features being derivatives of troponin, however clinical differentiators including biochemical results (white blood cell count, CRP, and estimated glomerular filtration rate [eGFR]) and age also have an effect.

#### Event prediction

The performance for the XGB-50 event prediction model in all training and testing data sets is summarized in Figure 2, and Supplemental Tables 1 and 5. XGB-50 had an AUC of 88.5% ± 0.5% in the overall testing data set (Figure 2E) and minor differences were observed when tested within the subsets of data set 1 and 2 (Supplemental Tables 2 and 3). These models also had low Brier scores (4.2% ± 0.1%) (Supplemental Tables 1 to 3).

Feature importance using normalized gain values is summarized in Figure 2I. The most important features appear to be biochemical data including minimum, maximum, and average troponin concentrations followed by albumin and eGFR as the 5 most important features. A large proportion of features are related to biochemical data although other clinical features such as age and ECG features were also present.

### Exploring model performance with lower repetition for clinical deployment

The XGB-50 and DL-50 diagnostic prediction models performed similarly when reduced to 19 and 5 repetitions, respectively. Similarly, the XGB-50 event prediction models were reduced to 9 repetitions with similar performance (Supplemental Tables 1 to 3, Supplemental Figure 1).

### Model calibration

In the diagnostic prediction models, the calibrated curves (Supplemental Figure 3) and statistics (Supplemental Table 12) for XGB-50 models demonstrate a tendency for underestimation of probabilities (calibration slope >1, intercept >0) which is better for level 2 compared to level 1 although the mean calibration is reasonable for both levels (2.1% and 3.3% respectively). These trends are also seen in the XGB-19 models.

The DL-50 models’ calibration curves (Supplemental Figure 3) and statistics appear to underestimate risk which is better for level 2 compared to level 1. The calibration statistics for level 1 suggests overall underestimation of risk and for level 2 suggests underestimation of low risk and overestimation of high risk with an overall trend toward underestimation (calibration slope <1, intercept >0, mean calibration >0). These trends are similar for the DL-5 models.

The event prediction model calibration curves (Supplemental Figure 3) demonstrate underestimation at lower probabilities with an overall trend toward underestimation (calibration slope >1, intercept >0, mean calibration >0) which is consistent with the XGB-9 models. The predicted probabilities for these models have narrow distributions as shown in Supplemental Figure 3.

### Sensitivity analyses

#### Importance of data availability on model performance

Diagnostic prediction model performance across both levels appear better with troponin-only data compared to clinical data only (age, gender, and prior comorbidity variables). The addition of clinical, ECG, and biochemistry (renal function, hemoglobin, and white blood cell count) data to troponin data, including when all input data are utilized, does not appear to improve model performance. Conversely, there appears to be a steady improvement in event prediction model performance with increasing input data (Figure 2J, Supplemental Figure 1J, Supplemental Table 8).

#### Sub-group analysis

The models performed consistently across age and where troponin data within the first 24 hours were restricted at set intervals except in subgroups with lower data points (>80 years and troponin data ≤3-hours from presentation). There were minor gender differences only seen in the event prediction models (Supplemental Figure 2, Supplemental Tables 9 to 11).

## Discussion

In this study, we outline the development of ML models with high predictive performance for diagnostic classification of myocardial injury and infarction phenotypes according to the Fourth UDMI and prediction of death or MI at 30 days in patients presenting to the ED with suspected ACS. Our prespecified performance targets were met in the XGB and DL diagnostic prediction models at level 1 and 2 and the event prediction XGB model. The clinical translation of these models into improvements in care and outcome is currently being prospectively tested within a cluster-randomized clinical trial. Models with lower repetition and thus faster processing time to allow real-time feedback at the clinical front-end were developed demonstrating similar performance. Troponin data were most important to the model performance with minor incremental improvement with the addition of clinical, ECG, and non-troponin pathology data—this was further highlighted when assessing feature importance.

Prespecified model performance goals were required to be achieved prior to deployment in clinical practice for ED decision support. Level 1 was designed to differentiate patients requiring admission vs consideration of early discharge from the ED. Misclassification of acute injury *pattern* as chronic injury *pattern* (false negative) may lead to discharge of patients requiring in-patient treatment hence our goal was to achieve a NPV ≥99% at level 1.^19^ Further differentiation of acute injury *pattern* into T1MI vs T2MI or acute nonischemic myocardial injury (level 2) is also important for consideration of an early invasive strategy and important ACS medications in T1MI. Misclassification of T1MI as T2MI/acute nonischemic myocardial injury (false negative) may lead to the withholding of evidence-based therapies although false positive misclassification may lead to invasive management of non-T1MI patients which could result in harm hence we maximized the NPV and PPV without a specific value. Similarly, this was also our goal for the event prediction model as limiting false negatives and false positives for death/MI at 30 days were of equal importance.

The importance of troponin data for model performance of XGB at level 1 and DL models for diagnostic prediction is expected given the differentiation of myocardial injury patterns according to the Fourth UDMI is directly related to the presence or absence of an acute rise/fall in troponin concentrations.^14^ Maximum troponin concentration remains the most important feature in level 2 for XGB models for diagnostic prediction however other pathology data including HbA1c, CRP, and hemoglobin also appear to be of relative importance (seen lower in the order for DL model feature importance as these models were not trained according to level 1 and 2). This is also expected given peak troponin concentrations are usually higher in T1MI vs T2MI or nonischemic myocardial injury; furthermore, T2MI is commonly provoked by conditions such as anemia, sepsis, or shock in which these pathology data would be expected to be abnormal if present.^21^, ^22^, ^23^, ^24^ HbA1c levels have also been associated with the severity of coronary artery disease in T1MI which may explain its presence as an important feature in the XGB level 2 models.^25^ Interestingly, ischemia on ECG was not an important feature for diagnostic prediction; however, myocardial ischemia is likely detected by the myriad of troponin-derived features. Furthermore, troponin data were available for all participants whereas ECG data were only available in data set 2 which may also explain this disparity.

Feature importance for the clinical event prediction model was related to pathology data, including troponin, which is unsurprising given the prediction of MI at 30 days. Other pathology data including albumin, eGFR, thyroid stimulating hormone, NT- pro brain natiuretic peptide, D-dimer, CRP and levels were also included, all of which have strong associations with mortality or MI in prior studies.^26^, ^27^, ^28^, ^29^, ^30^

Calibration is an important aspect of risk prediction models to ensure generalizability.^31^ There was a general trend toward underestimation of predictive probabilities for diagnostic and event prediction models. In the diagnostic prediction models, the DL models appeared to be better calibrated than XGB. This is likely due to XGB “pushing” predictive probabilities toward the extremes. In the event prediction models, calibration led to skewing of the predicted probability distribution likely because of class imbalance in a binary classifier. The strength of the event prediction models is in low-risk probabilities where it demonstrates good calibration. The ability to accurately identify low-risk suspected ACS patients in the ED suitable for discharge is hence advantageous for clinical implementation.^19^

Applications of AI and ML have been widely used within health care and in cardiovascular medicine in recent years with the increasing availability of big data and EHR. ML has been utilized within cardiovascular medicine for interpretation of ECG,^32^^,^^33^ imaging modalities such as echocardiography, computed tomography and magnetic resonance imaging,^34^, ^35^, ^36^, ^37^ diagnosis,^38^, ^39^, ^40^, ^41^, ^42^, ^43^ and prognosis.^44^^,^^45^ A systematic review by Stewart et al^46^ reported applications of ML in the assessment of suspected ACS in the ED showing most studies reported the diagnostic performance for MI and prognostic performance for major adverse cardiovascular events however no studies reported the differentiation of index presentations into myocardial injury and infarction. The advantage of our ML models is the ability to phenotype both MI (T1MI and T2MI) and myocardial injury (acute and chronic injury) as well as the ability to predict death or MI at 30 days.

Despite the development of numerous applications of ML, very few have been assessed in clinical practice or randomized clinical trials.^35^^,^^38^^,^^46^^,^^47^ A recent systematic review of RCTs of ML interventions in health care found only 41 RCTs across all specialties; only 3 were in the field of cardiology and 2 of these studies had <100 patients in their data set. We plan to externally validate our diagnostic and event prediction models in a cluster-randomized, multicenter clinical trial called ‘RAPIDx AI’ where the ML models will be embedded within a clinical decision support system for the assessment of suspected ACS patients within the ED ACTRN12620001319965). The trial design and protocol for the RAPIDx AI clinical trial will be published separately. Importantly, the data sets used to build our models are predominantly from EHR—the training data sets are from 6 metropolitan hospitals however the testing data set included patients from 26 hospitals in the same state where the clinical trial will take place. Furthermore, to mitigate the risks of data set shift, we trained the models on 2 data sets—1 of which (data set 2) included contemporary data between 2020 and 2021.^48^^,^^49^ Although several feature variables were used for the final model, our sensitivity analysis demonstrate that only clinical and troponin data are required for the diagnostic prediction model to achieve high predictive performance, hence not all input data are required during “real-world” implementation.

### Study Limitations

First, although clinical adjudications were performed using the Fourth UDMI with clear instructions provided, it was still at the discretion of the adjudicating clinicians hence the possibility of measurement bias. Second, there were missing non-troponin pathology data that had not been requested in clinical practice—this is however reflective of the routine clinical environment and EHR data. Test ordering was at the discretion of treating physicians (Supplemental Table 6). As shown in our sensitivity analyses, these results are not required for the model to function, and their availability is only expected when there is a clinical indication. Third, we did not have raw ECG data to incorporate into the model hence the automated tracing interpretation was used to code the 12 binary variables. This may be advantageous when deploying the models within the clinical environment since the real-time integration of raw ECG data with the model may be challenging, though represents a future opportunity with increasing digital maturation of health systems. Fourth, clinical symptoms were not available as features within the training/testing data sets hence were not integrated into the model although this will be collected prospectively in the clinical trial for future improvements. Fifth, we did not include the study site as a covariate in the models although we do not observe significant differences in the results when comparing test performance in the overall test set (26 study sites) and within data sets 1 (6 study sites) and 2 (1 study site). Sixth, the 30-day event rates were low (<5%) hence event predictive performance may be limited due to class imbalance.

## Conclusions

Using various large data sets predominantly from EHR data, we have developed ML models to support ED diagnosis of MI and myocardial injury as well as 30-day risk evaluation for ED patients with suspected ACS which met our prespecified performance goals for clinical deployment. These models will be externally validated in a prospective, multicenter cluster-randomized clinical trial evaluating the impact of ML models in clinical practice on care, clinical outcomes, and cost-effectiveness.

Perspectives **COMPETENCY IN MEDICAL KNOWLEDGE AND PATIENT CARE:** ML models using data predominantly from EHR can be developed for diagnostic classification of myocardial injury and infarction phenotypes and prediction of 30-day death/MI in patients presenting with suspected ACS. The models have predictive performance such that they could be deployed in clinical practice as a clinical decision support system.

**TRANSLATIONAL OUTLOOK:** These models require external validation in a prospective, multicenter cluster randomized clinical trial evaluating the impact of ML models in clinical practice on care, clinical outcomes, and cost-effectiveness.

## Funding support and author disclosures

Supported by NHMRC MRFF Partnership Grant APP1191914. Dr Chew has received grant in aid from Roche Diagnostics and Edwards Lifesciences. Dr Cullen has received research grants from Abbott Diagnostics, Siemens, Beckman Coulter and speaker honoraria from Siemens, Abbott Diagnostics, Novartis, AstraZeneca. All other authors have reported that they have no relationships relevant to the contents of this paper to disclose.
