# Supplementary material for: Machine-Learning for Phenotyping and Prognostication of Myocardial Infarction and Injury in Suspected Acute Coronary Syndrome
Source: JACC Adv. 2024 Jun 19;3(9):101011. doi: 10.1016/j.jacadv.2024.101011 (PMC11450946; doi:10.1016/j.jacadv.2024.101011)
Supplement: Supplementary data [file mmc1.docx]

**Supplemental Appendix**

**Supplemental methods**

*Datasets:* A total of 3 datasets were used in this study.

Dataset 1: The available data was a combination of two randomized controlled trials (RCTs) and EHR data. Study A was a high sensitivity cardiac troponin study (n=1937, July 2011-March 2013) and Study B was the RAPID TnT trial (n=3288, August 2015-April 2019). Both study designs have been described previously. (1,2) EHR data (n=1532) was included to improve generalisability of this dataset beyond RCT data. This was a random sample of patients presenting to the ED of 6 metropolitan hospitals in Adelaide, South Australia between June 2011 and September 2019 with at least 1 hs-cTnT concentration measurement.

Dataset 2: This dataset included a random sample derived from EHR data (n=5248) of patients presenting to the ED of a metropolitan hospital in Adelaide, South Australia from 17^th^ February 2020 through 18^th^ February 2021 where at least 1 hs-cTnT concentration was measured. This dataset additionally included ECG data which was extracted via the local ECG management system for all randomly selected participants hence there was no confounding reason for the availability of ECG data. ECG meta-data were analysed for the sub-text ECG interpretation visible on the tracing. We categorized this into 12 binary variables using a free-text search function. (Supplemental Table 5) This data was available for all patients in this dataset.

Dataset 3: This dataset included a random sample derived from EHR data (n=6,627) of patients presenting to the ED of 26 hospitals across South Australia between 17^th^ February 2020 and 18^th^ February 2021 where at least 1 hs-cTnT concentrations was measured.

Random sampling of EHR data was performed to collect a representative sample from the database for clinical adjudication.

*Applying the Fourth Universal definition of MI*

All participants with elevated troponin concentrations (above the 99^th^ percentile) were assessed for the following:

1) Evidence for a rise and/or fall of hs-cTnT of $>$20% delta AND $\geq$2.5ng/L/hr change

2) A ‘clear culprit’ on angiography OR a regional wall motion abnormality

on myocardial imaging OR dynamic ECG changes [ST segment deviation

$\geq$0.5mm in 2 contiguous leads or dynamic T wave inversion in leads V1-4]

3) A clear ischaemic history

- Events meeting criteria 1 and 2 or 3 were labelled with MI. If there was a clear precipitating cause, the event was labelled Type 2 MI, as opposed to Type 1 MI.
- Those meeting criteria 1, but not criteria 2 or 3 was deemed “acute non-ischaemic myocardial injury”
- Those not meeting criteria 1 were labelled “chronic myocardial injury” if the clinical presentation was consistent with this diagnosis

*Definition of index MI and 30-day death/MI*

MI diagnosed within 24 hours of presentation among participants continuously in-hospital was considered as the index presenting MI and not included as an outcome MI. An MI documented to have commenced outside this time (recurrent MI), or within 24 hours of presentation among participants already discharged from the hospital (missed MI), were considered an outcome MI. All-cause mortality within 30-days of the index presentation was considered as an outcome.

*Data pre-processing and transformation*

1. Handling of missing data: When the value of an input feature is missing, the XGB model automatically finds a default direction for the corresponding tree node, and such default direction is learned during the tree construction process which optimizes the training loss. For the DL model, we used the mean substitution if the value of an input feature was missing. Specifically, for non-troponin pathology data with missing values (not requested), default values within the normal reference range were used.
2. Feature engineering/extraction/selection: To standardize the troponin data across the datasets, we computed 10 variables to quantify the troponin profile during the first 24-hours from presentation: logarithm of the first troponin concentration, average value of the troponin concentrations (of all available in the first 24-hours), average velocity of troponin change (calculated by difference in troponin concentrations divided by time), maximum/minimum troponin concentration and the difference between the concentrations, maximum/minimum velocity of troponin change and the difference between the concentrations as well as a quotient of maximum troponin concentration divided by minimum troponin concentration (“divtrop” in Figure 2H and Supplemental Figure 1H).

No feature selection was explicitly given as the supervised learning methods learnt to select relevant features.

1. Duplicate participants: Datasets were assessed to ensure there were no duplicate participants across all datasets.

*Defining ML analysis architecture and ML methods*

1. Training protocol and evaluation:

The datasets were first split into three subsets: training (Dataset 1: 3,574; Dataset 2: 3,148), validation (Dataset 1: 1,192; Dataset 2: 1,050), and testing (Dataset 1: 1,192; Dataset 2: 1,050; Dataset 3: 6,627). For the XGB model, we used repeated and stratified 5-fold cross validation on a combined set including the training and validation subsets. For the DL model, due to a longer training time, we used the training set to train the DL model and validate on the validation set. The performance of both models was reported on the same test set. To reduce the performance variation due to random dataset splits and model initialisation, we repeated testing up to a maximum of 50 times with different dataset splits. We also reported results for models with the least number of repetitions required to achieve AUC within 1% of the maximum repetition models in order to shorten real-time feedback when the models are deployed within the clinical environment.

1. Method including method-specific hyperparameters, optimization strategies and generalization techniques:

For the XGB model, we used the gbtree booster and 200 boosting rounds. The loss function of the XGB model was set to a binary logistic regression type otherwise the training followed the default XGB training. For the DL model, the architecture was a two-layer structure with 512 Batch Normalized Rectified Linear Units (ReLU) units. (3,4) Dropout layers were applied after each layer to improve the generalisation of the neural network. (5) The training of the MLP starts with a learning rate of 5e-3 and linearly reduced to 1/100 of the initial learning rate over the course of 100 training epochs (an epoch iterates through all training samples once). The optimizer of the MLP network was the standard stochastic gradient descent (SGD) optimizer with a batch size of 128 samples per training iteration. The training of the MLP takes advantage of CUDA-enabled GPU computation offered by PyTorch. (6)

1. Threshold methods:

The models (both XGB and DL) by default use 0.5 as threshold when computing the level 1 and level 2 performance as each layer is a binary classification. There were two threshold methods used to choose a different threshold (other than 0.5): 1) optimized towards receiver operating characteristic [ROC] curve; 2) optimized towards a target true positive rate [TPR] of 0.99.

*Computational architecture*:

The processor used was an i7-8700 6-core 12 threads with 3.20 GHz base frequency, 32 GB of memory using Python 3.9. The following software were used: NumPy, SciPy, scikit-learn, XGBoost, PyTorch.

|  | Average sensitivity ± 95% CI | | Average specificity ± 95% CI | | | Average PPV ± 95% CI | | | Average NPV ± 95% CI | | | | Average AUC ± 95% CI | | | Average Brier Score ± 95% CI | |
| --- | --- | --- | --- | --- | --- | --- | --- | --- | --- | --- | --- | --- | --- | --- | --- | --- | --- |
| **Diagnostic prediction, level 1** | Training | Testing | | Training | Testing | | Training | Testing | | Training | Testing | Training | | Testing | Training | | Testing |
| DL-50 (threshold: default) | 92.7% ± 2.9% | 83.0% ± 3.4% | | 98.2% ± 0.6% | 98.7% ± 0.4% | | 92.3% ± 2.2% | 95.5% ± 1.2% | | 98.4% ± 0.6% | 94.7% ± 1.0% | 99.5% ± 0.0% | | 98.8% ± 0.2% | 2.1% ± 0.1% | | 3.9% ± 0.2% |
| DL-5 (threshold: default) | 92.3% ± 2.4% | 81.9% ± 3.1% | | 98.5% ± 0.4% | 98.9% ± 0.3% | | 93.4% ± 1.5% | 96.0% ± 0.8% | | 98.3% ± 0.5% | 94.3% ± 0.9% | 99.5% ± 0.0% | | 98.8% ± 0.1% | 2.1% ± 0.1% | | 3.9% ± 0.1% |
| DL-50 (threshold: ROC) | 99.0% ± 0.5% | 92.5% ± 1.4% | | 94.6% ± 1.2% | 96.4% ± 0.7% | | 80.6% ± 3.5% | 89.5% ± 1.6% | | 99.8% ± 0.1% | 97.5% ± 0.4% | 99.5% ± 0.0% | | 98.8% ± 0.2% | 2.1% ± 0.1% | | 3.9% ± 0.2% |
| DL-5 (threshold: ROC) | 99.0% ± 0.3% | 92.3% ± 1.4% | | 94.7% ± 1.4% | 96.6% ± 0.7% | | 81.2% ± 4.2% | 89.9% ± 1.5% | | 99.8% ± 0.1% | 97.5% ± 0.4% | 99.5% ± 0.0% | | 98.8% ± 0.1% | 2.1% ± 0.1% | | 3.9% ± 0.1% |
| DL-50 (threshold: TPR) | 99.9% ± 0.1% | 97.8% ± 0.7% | | 81.4% ± 5.2% | 90.0% ± 2.3% | | 55.6% ± 6.3% | 76.6% ± 3.8% | | 100.0% ± 0.0% | 99.2% ± 0.3% | 99.5% ± 0.0% | | 98.8% ± 0.2% | 2.1% ± 0.1% | | 3.9% ± 0.2% |
| DL-5 (threshold: TPR) | 99.9% ± 0.0% | 97.5% ± 0.5% | | 83.1% ± 1.8% | 90.8% ± 1.0% | | 57.3% ± 3.1% | 77.9% ± 1.7% | | 100.0% ± 0.0% | 99.1% ± 0.2% | 99.5% ± 0.0% | | 98.8% ± 0.1% | 2.1% ± 0.1% | | 3.9% ± 0.1% |
| XGB-50 (threshold: default) | 97.1% ± 0.8% | 83.9% ± 2.3% | | 99.7% ± 0.1% | 99.3% ± 0.2% | | 98.6% ± 0.6% | 97.6% ± 0.6% | | 99.3% ± 0.2% | 94.9% ± 0.7% | 99.9% ± 0.0% | | 99.2% ± 0.1% | 1.0% ± 0.1% | | 3.6% ± 0.2% |
| XGB-19 (threshold: default) | 97.0% ± 1.0% | 83.7% ± 2.5% | | 99.7% ± 0.2% | 99.3% ± 0.2% | | 98.6% ± 0.7% | 97.7% ± 0.6% | | 99.3% ± 0.2% | 94.9% ± 0.7% | 99.9% ± 0.0% | | 99.2% ± 0.1% | 1.1% ± 0.1% | | 3.6% ± 0.2% |
| XGB-50 (threshold: ROC) | 99.1% ± 0.2% | 92.5% ± 1.2% | | 97.7% ± 0.6% | 97.9% ± 0.4% | | 90.8% ± 2.3% | 93.6% ± 1.2% | | 99.8% ± 0.1% | 97.5% ± 0.4% | 99.9% ± 0.0% | | 99.2% ± 0.1% | 1.0% ± 0.1% | | 3.6% ± 0.2% |
| XGB-19 (threshold: ROC) | 99.1% ± 0.2% | 92.3% ± 1.1% | | 97.9% ± 0.5% | 98.0% ± 0.4% | | 91.3% ± 2.0% | 93.9% ± 1.0% | | 99.8% ± 0.1% | 97.5% ± 0.3% | 99.9% ± 0.0% | | 99.2% ± 0.1% | 1.1% ± 0.1% | | 3.6% ± 0.2% |
| XGB-50 (threshold: TPR) | 99.9% ± 0.1% | 97.7% ± 0.7% | | 89.5% ± 2.1% | 93.5% ± 1.1% | | 68.5% ± 4.1% | 83.3% ± 2.2% | | 100.0% ± 0.0% | 99.2% ± 0.2% | 99.9% ± 0.0% | | 99.2% ± 0.1% | 1.0% ± 0.1% | | 3.6% ± 0.2% |
| XGB-19 (threshold: TPR) | 100.0% ± 0.1% | 97.8% ± 0.7% | | 89.0% ± 2.4% | 93.3% ± 1.2% | | 67.5% ± 4.6% | 82.9% ± 2.4% | | 100.0% ± 0.0% | 99.2% ± 0.3% | 99.9% ± 0.0% | | 99.2% ± 0.1% | 1.1% ± 0.1% | | 3.6% ± 0.2% |
|  |  |  | |  |  | |  |  | |  |  |  | |  |  | |  |
| **Diagnostic prediction, level 2** | Average sensitivity ± 95% CI | | | Average specificity ± 95% CI | | | Average PPV ± 95% CI | | | Average NPV ± 95% CI | | Average AUC ± 95% CI | | | Average Brier Score ± 95% CI | | |
| DL-50 (threshold: default) | 81.3% ± 6.9% | 36.6% ± 5.2% | | 99.7% ± 0.2% | 99.0% ± 0.4% | | 94.4% ± 2.5% | 77.9% ± 4.4% | | 98.9% ± 0.4% | 94.5% ± 0.4% | 99.8% ± 0.0% | | 94.6% ± 0.9% | 1.1% ± 0.1% | | 4.7% ± 0.2% |
| DL-5 (threshold: default) | 79.5% ± 6.0% | 35.8% ± 6.6% | | 99.7% ± 0.1% | 99.1% ± 0.4% | | 94.8% ± 2.1% | 79.4% ± 4.1% | | 98.8% ± 0.4% | 94.4% ± 0.5% | 99.8% ± 0.0% | | 93.8% ± 1.9% | 1.1% ± 0.1% | | 4.7% ± 0.2% |
| DL-50 (threshold: ROC) | 99.9% ± 0.2% | 79.5% ± 5.8% | | 93.3% ± 2.4% | 92.2% ± 1.9% | | 48.9% ± 8.7% | 48.7% ± 4.3% | | 100.0% ± 0.0% | 98.0% ± 0.5% | 99.8% ± 0.0% | | 94.6% ± 0.9% | 1.1% ± 0.1% | | 4.7% ± 0.2% |
| DL-5 (threshold: ROC) | 99.9% ± 0.1% | 80.2% ± 5.9% | | 92.8% ± 2.7% | 91.9% ± 2.1% | | 47.7% ± 8.6% | 48.1% ± 5.1% | | 100.0% ± 0.0% | 98.1% ± 0.5% | 99.8% ± 0.0% | | 93.8% ± 1.9% | 1.1% ± 0.1% | | 4.7% ± 0.2% |
| DL-50 (threshold: TPR) | 100.0% ± 0.0% | 95.7% ± 3.7% | | 73.1% ± 11.9% | 78.3% ± 7.4% | | 21.0% ± 7.6% | 30.3% ± 6.4% | | 100.0% ± 0.0% | 99.5% ± 0.4% | 99.8% ± 0.0% | | 94.6% ± 0.9% | 1.1% ± 0.1% | | 4.7% ± 0.2% |
| DL-5 (threshold: TPR) | 100.0% ± 0.0% | 93.0% ± 6.6% | | 74.9% ± 15.3% | 80.1% ± 9.7% | | 25.2% ± 12.4% | 33.1% ± 9.3% | | 100.0% ± 0.0% | 99.3% ± 0.6% | 99.8% ± 0.0% | | 93.8% ± 1.9% | 1.1% ± 0.1% | | 4.7% ± 0.2% |
| XGB-50 (threshold: default) | 80.8% ± 7.5% | 33.1% ± 5.7% | | 100.0% ± 0.0% | 99.2% ± 0.3% | | 99.3% ± 0.5% | 80.0% ± 4.5% | | 98.8% ± 0.4% | 94.2% ± 0.5% | 99.9% ± 0.0% | | 95.5% ± 0.2% | 1.0% ± 0.1% | | 4.6% ± 0.1% |
| XGB-19 (threshold: default) | 80.2% ± 7.6% | 32.6% ± 5.7% | | 100.0% ± 0.0% | 99.3% ± 0.3% | | 99.4% ± 0.5% | 80.8% ± 4.8% | | 98.8% ± 0.5% | 94.2% ± 0.4% | 99.9% ± 0.0% | | 95.5% ± 0.3% | 1.0% ± 0.1% | | 4.6% ± 0.1% |
| XGB-50 (threshold: ROC) | 99.9% ± 0.1% | 85.2% ± 3.2% | | 94.0% ± 1.2% | 90.6% ± 1.2% | | 50.9% ± 5.0% | 45.5% ± 2.4% | | 100.0% ± 0.0% | 98.5% ± 0.3% | 99.9% ± 0.0% | | 95.5% ± 0.2% | 1.0% ± 0.1% | | 4.6% ± 0.1% |
| XGB-19 (threshold: ROC) | 99.9% ± 0.1% | 85.8% ± 3.3% | | 93.7% ± 1.3% | 90.4% ± 1.3% | | 49.6% ± 5.0% | 44.9% ± 2.5% | | 100.0% ± 0.0% | 98.6% ± 0.3% | 99.9% ± 0.0% | | 95.5% ± 0.3% | 1.0% ± 0.1% | | 4.6% ± 0.1% |
| XGB-50 (threshold: TPR) | 100.0% ± 0.0% | 95.7% ± 1.6% | | 85.2% ± 3.8% | 83.3% ± 2.5% | | 29.9% ± 4.8% | 34.6% ± 2.9% | | 100.0% ± 0.0% | 99.5% ± 0.2% | 99.9% ± 0.0% | | 95.5% ± 0.2% | 1.0% ± 0.1% | | 4.6% ± 0.1% |
| XGB-19 (threshold: TPR) | 100.0% ± 0.0% | 95.6% ± 1.6% | | 84.8% ± 4.2% | 83.1% ± 2.6% | | 29.6% ± 5.1% | 34.3% ± 2.9% | | 100.0% ± 0.0% | 99.5% ± 0.2% | 99.9% ± 0.0% | | 95.5% ± 0.3% | 1.0% ± 0.1% | | 4.6% ± 0.1% |
|  |  |  | |  |  | |  |  | |  |  |  | |  |  | |  |
| **Event prediction** | Average sensitivity ± 95% CI | | | Average specificity ± 95% CI | | | Average PPV ± 95% CI | | | Average NPV ± 95% CI | | Average AUC ± 95% CI | | | Average Brier Score ± 95% CI | | |
| XGB-50 (threshold: default) | 0.0% ± 0.0% | 0.0% ± 0.0% | | 100.0% ± 0.0% | 100.0% ± 0.0% | | 0.0% ± 0.0% | 0.0% ± 0.0% | | 95.7% ± 0.1% | 94.9% ± 0.1% | 96.8% ± 0.7% | | 88.5% ± 0.5% | 3.3% ± 0.1% | | 4.2% ± 0.1% |
| XGB-9 (threshold: default) | 0.0% ± 0.0% | 0.0% ± 0.0% | | 100.0% ± 0.0% | 100.0% ± 0.0% | | 0.0% ± 0.0% | 0.0% ± 0.0% | | 95.7% ± 0.1% | 94.9% ± 0.1% | 96.9% ± 0.9% | | 88.4% ± 0.8% | 3.3% ± 0.1% | | 4.3% ± 0.1% |
| XGB-50 (threshold: ROC) | 96.9% ± 1.3% | 84.9% ± 3.8% | | 77.9% ± 4.5% | 78.2% ± 3.2% | | 17.0% ± 2.8% | 17.5% ± 1.6% | | 99.8% ± 0.1% | 99.0% ± 0.2% | 96.8% ± 0.7% | | 88.5% ± 0.5% | 3.3% ± 0.1% | | 4.2% ± 0.1% |
| XGB-9 (threshold: ROC) | 97.9% ± 0.9% | 87.3% ± 3.5% | | 74.4% ± 4.8% | 75.5% ± 3.5% | | 15.0% ± 2.7% | 16.3% ± 1.6% | | 99.9% ± 0.0% | 99.1% ± 0.2% | 96.9% ± 0.9% | | 88.4% ± 0.8% | 3.3% ± 0.1% | | 4.3% ± 0.1% |
| XGB-50 (threshold: TPR) | 100.0% ± 0.0% | 96.5% ± 1.3% | | 30.3% ± 6.1% | 54.5% ± 4.3% | | 6.1% ± 0.5% | 10.3% ± 0.8% | | 100.0% ± 0.0% | 99.7% ± 0.1% | 100.0% ± 0.0% | | 88.6% ± 0.7% | 0.7% ± 0.2% | | 4.1% ± 0.1% |
| XGB-9 (threshold: ROC) | 100.0% ± 0.0% | 96.3% ± 1.4% | | 30.4% ± 5.7% | 55.0% ± 3.8% | | 6.1% ± 0.5% | 10.4% ± 0.7% | | 100.0% ± 0.0% | 99.7% ± 0.1% | 100.0% ± 0.0% | | 88.4% ± 0.6% | 0.7% ± 0.2% | | 4.1% ± 0.1% |

Supplemental Table 1 | Model performance metrics in the overall training and testing datasets

AUC: Area under curve; CI: confidence interval; DL: deep learning model; NPV: negative predictive value; PPV: positive predictive value; ROC: receiver operating characteristic; TPR: true positive rate; XGB: eXtreme gradient boosting

|  | Average sensitivity ± 95% CI | | Average specificity ± 95% CI | | | Average PPV ± 95% CI | | | Average NPV ± 95% CI | | | Average AUC ± 95% CI | | | Average Brier Score ± 95% CI | | |
| --- | --- | --- | --- | --- | --- | --- | --- | --- | --- | --- | --- | --- | --- | --- | --- | --- | --- |
| **Diagnostic prediction, level 1** | Training | Testing | | Training | Testing | | Training | Testing | | Training | Testing | | Training | Testing | | Training | Testing |
| DL-50 (threshold: default) | 93.2% ± 2.5% | 88.2% ± 3.0% | | 97.6% ± 0.7% | 96.7% ± 0.9% | | 89.0% ± 2.9% | 84.6% ± 3.5% | | 98.6% ± 0.5% | 97.6% ± 0.6% | | 99.4% ± 0.1% | 98.5% ± 0.3% | | 2.4% ± 0.1% | 3.4% ± 0.4% |
| DL-5 (threshold: default) | 93.0% ± 1.9% | 85.4% ± 3.3% | | 98.0% ± 0.5% | 97.0% ± 0.8% | | 90.3% ± 1.8% | 85.5% ± 3.5% | | 98.6% ± 0.4% | 96.9% ± 0.8% | | 99.4% ± 0.1% | 98.4% ± 0.2% | | 2.3% ± 0.1% | 3.6% ± 0.3% |
| DL-50 (threshold: ROC) | 98.6% ± 0.5% | 95.8% ± 1.7% | | 93.6% ± 1.2% | 92.7% ± 1.3% | | 76.1% ± 3.5% | 72.9% ± 3.5% | | 99.7% ± 0.1% | 99.1% ± 0.4% | | 99.4% ± 0.1% | 98.5% ± 0.3% | | 2.4% ± 0.1% | 3.4% ± 0.4% |
| DL-5 (threshold: ROC) | 98.6% ± 0.2% | 95.1% ± 2.0% | | 93.8% ± 1.5% | 92.8% ± 1.2% | | 76.6% ± 4.2% | 73.5% ± 2.1% | | 99.7% ± 0.0% | 98.9% ± 0.4% | | 99.4% ± 0.1% | 98.4% ± 0.2% | | 2.3% ± 0.1% | 3.6% ± 0.3% |
| DL-50 (threshold: TPR) | 99.8% ± 0.2% | 98.8% ± 0.8% | | 83.7% ± 3.6% | 83.2% ± 3.6% | | 56.1% ± 5.1% | 54.9% ± 5.1% | | 100.0% ± 0.0% | 99.7% ± 0.2% | | 99.4% ± 0.1% | 98.5% ± 0.3% | | 2.4% ± 0.1% | 3.4% ± 0.4% |
| DL-5 (threshold: TPR) | 99.8% ± 0.1% | 98.7% ± 0.2% | | 84.9% ± 1.5% | 84.5% ± 1.4% | | 57.5% ± 2.9% | 57.0% ± 2.6% | | 100.0% ± 0.0% | 99.7% ± 0.1% | | 99.4% ± 0.1% | 98.4% ± 0.2% | | 2.3% ± 0.1% | 3.6% ± 0.3% |
| XGB-50 (threshold: default) | 95.8% ± 0.9% | 90.5% ± 2.2% | | 99.6% ± 0.2% | 97.9% ± 0.5% | | 97.8% ± 0.9% | 89.9% ± 2.3% | | 99.2% ± 0.2% | 98.1% ± 0.5% | | 99.9% ± 0.0% | 99.0% ± 0.2% | | 1.2% ± 0.1% | 2.6% ± 0.3% |
| XGB-19 (threshold: default) | 95.6% ± 1.1% | 90.2% ± 2.6% | | 99.5% ± 0.2% | 98.0% ± 0.5% | | 97.7% ± 0.9% | 90.2% ± 2.2% | | 99.1% ± 0.2% | 98.0% ± 0.5% | | 99.9% ± 0.0% | 99.0% ± 0.2% | | 1.2% ± 0.1% | 2.6% ± 0.3% |
| XGB-50 (threshold: ROC) | 98.3% ± 0.4% | 95.1% ± 1.4% | | 97.4% ± 0.7% | 95.6% ± 0.9% | | 88.6% ± 2.7% | 81.4% ± 3.3% | | 99.7% ± 0.1% | 99.0% ± 0.3% | | 99.9% ± 0.0% | 99.0% ± 0.2% | | 1.2% ± 0.1% | 2.6% ± 0.3% |
| XGB-19 (threshold: ROC) | 98.3% ± 0.4% | 94.9% ± 1.5% | | 97.6% ± 0.6% | 95.8% ± 0.7% | | 89.2% ± 2.2% | 82.0% ± 2.9% | | 99.6% ± 0.1% | 98.9% ± 0.3% | | 99.9% ± 0.0% | 99.0% ± 0.2% | | 1.2% ± 0.1% | 2.6% ± 0.3% |
| XGB-50 (threshold: TPR) | 99.9% ± 0.1% | 98.0% ± 0.9% | | 89.0% ± 1.9% | 88.4% ± 2.1% | | 65.3% ± 3.8% | 63.4% ± 4.5% | | 100.0% ± 0.0% | 99.5% ± 0.2% | | 99.9% ± 0.0% | 99.0% ± 0.2% | | 1.2% ± 0.1% | 2.6% ± 0.3% |
| XGB-19 (threshold: TPR) | 99.9% ± 0.1% | 97.8% ± 0.9% | | 88.5% ± 2.2% | 88.0% ± 2.2% | | 64.4% ± 4.2% | 62.5% ± 4.6% | | 100.0% ± 0.0% | 99.5% ± 0.2% | | 99.9% ± 0.0% | 99.0% ± 0.2% | | 1.2% ± 0.1% | 2.6% ± 0.3% |
|  |  |  | |  |  | |  |  | |  |  | |  |  | |  |  |
| **Diagnostic prediction, level 2** | Average sensitivity ± 95% CI | | | Average specificity ± 95% CI | | | Average PPV ± 95% CI | | | Average NPV ± 95% CI | | | Average AUC ± 95% CI | | | Average Brier Score ± 95% CI | |
| DL-50 (threshold: default) | 71.4% ± 8.6% | 58.0% ± 8.9% | | 99.6% ± 0.2% | 98.9% ± 0.5% | | 89.9% ± 4.2% | 73.3% ± 8.6% | | 98.6% ± 0.4% | 97.9% ± 0.5% | | 99.5% ± 0.1% | 96.8% ± 1.1% | | 1.3% ± 0.1% | 2.3% ± 0.3% |
| DL-5 (threshold: default) | 69.9% ± 5.6% | 54.9% ± 12.2% | | 99.6% ± 0.2% | 99.0% ± 0.4% | | 90.6% ± 3.2% | 73.0% ± 6.4% | | 98.5% ± 0.3% | 97.8% ± 0.6% | | 99.5% ± 0.1% | 96.7% ± 1.0% | | 1.3% ± 0.1% | 2.4% ± 0.4% |
| DL-50 (threshold: ROC) | 99.7% ± 0.4% | 90.6% ± 4.9% | | 92.2% ± 2.6% | 91.5% ± 2.3% | | 40.6% ± 7.9% | 35.8% ± 6.0% | | 100.0% ± 0.0% | 99.5% ± 0.3% | | 99.5% ± 0.1% | 96.8% ± 1.1% | | 1.3% ± 0.1% | 2.3% ± 0.3% |
| DL-5 (threshold: ROC) | 99.8% ± 0.3% | 91.1% ± 6.6% | | 91.8% ± 3.0% | 90.8% ± 3.3% | | 40.2% ± 8.6% | 34.5% ± 7.4% | | 100.0% ± 0.0% | 99.5% ± 0.4% | | 99.5% ± 0.1% | 96.7% ± 1.0% | | 1.3% ± 0.1% | 2.4% ± 0.4% |
| DL-50 (threshold: TPR) | 100.0% ± 0.0% | 97.9% ± 2.2% | | 72.8% ± 10.6% | 72.4% ± 10.9% | | 17.3% ± 5.9% | 16.9% ± 6.0% | | 100.0% ± 0.0% | 99.9% ± 0.1% | | 99.5% ± 0.1% | 96.8% ± 1.1% | | 1.3% ± 0.1% | 2.3% ± 0.3% |
| DL-5 (threshold: TPR) | 100.0% ± 0.0% | 97.9% ± 2.0% | | 75.5% ± 12.8% | 75.1% ± 12.8% | | 20.9% ± 9.0% | 19.6% ± 8.3% | | 100.0% ± 0.0% | 99.9% ± 0.1% | | 99.5% ± 0.1% | 96.7% ± 1.0% | | 1.3% ± 0.1% | 2.4% ± 0.4% |
| XGB-50 (threshold: default) | 74.1% ± 8.8% | 49.1% ± 10.2% | | 99.9% ± 0.0% | 99.4% ± 0.3% | | 98.5% ± 1.0% | 80.0% ± 7.0% | | 98.7% ± 0.4% | 97.5% ± 0.6% | | 99.9% ± 0.0% | 97.7% ± 0.7% | | 1.1% ± 0.1% | 2.2% ± 0.3% |
| XGB-19 (threshold: default) | 73.3% ± 8.6% | 47.9% ± 9.4% | | 99.9% ± 0.0% | 99.4% ± 0.3% | | 98.7% ± 1.0% | 79.4% ± 8.8% | | 98.7% ± 0.4% | 97.5% ± 0.6% | | 99.9% ± 0.0% | 97.7% ± 0.7% | | 1.1% ± 0.1% | 2.2% ± 0.3% |
| XGB-50 (threshold: ROC) | 100.0% ± 0.1% | 93.2% ± 3.2% | | 93.5% ± 1.1% | 92.6% ± 1.3% | | 44.0% ± 4.3% | 39.2% ± 5.1% | | 100.0% ± 0.0% | 99.6% ± 0.2% | | 99.9% ± 0.0% | 97.7% ± 0.7% | | 1.1% ± 0.1% | 2.2% ± 0.3% |
| XGB-19 (threshold: ROC) | 100.0% ± 0.2% | 93.6% ± 3.4% | | 93.2% ± 1.1% | 92.4% ± 1.6% | | 43.0% ± 4.1% | 37.8% ± 5.6% | | 100.0% ± 0.0% | 99.7% ± 0.2% | | 99.9% ± 0.0% | 97.7% ± 0.7% | | 1.1% ± 0.1% | 2.2% ± 0.3% |
| XGB-50 (threshold: TPR) | 100.0% ± 0.0% | 97.5% ± 2.4% | | 85.4% ± 3.4% | 85.0% ± 3.2% | | 26.3% ± 4.1% | 25.3% ± 4.4% | | 100.0% ± 0.0% | 99.9% ± 0.1% | | 99.9% ± 0.0% | 97.7% ± 0.7% | | 1.1% ± 0.1% | 2.2% ± 0.3% |
| XGB-19 (threshold: TPR) | 100.0% ± 0.0% | 97.8% ± 2.0% | | 85.0% ± 3.7% | 84.6% ± 3.5% | | 26.1% ± 4.4% | 24.0% ± 4.2% | | 100.0% ± 0.0% | 99.9% ± 0.1% | | 99.9% ± 0.0% | 97.7% ± 0.7% | | 1.1% ± 0.1% | 2.2% ± 0.3% |
|  |  |  | |  |  | |  |  | |  |  | |  |  | |  |  |
| **Event prediction** | Average sensitivity ± 95% CI | | | Average specificity ± 95% CI | | | Average PPV ± 95% CI | | | Average NPV ± 95% CI | | | Average AUC ± 95% CI | | | Average Brier Score ± 95% CI | |
| XGB-50 (threshold: default) | 0.0% ± 0.0% | 0.0% ± 0.0% | | 100.0% ± 0.0% | 100.0% ± 0.0% | | 0.0% ± 0.0% | 0.0% ± 0.0% | | 96.1% ± 0.1% | 96.0% ± 0.6% | | 96.4% ± 0.7% | 86.8% ± 2.3% | | 3.0% ± 0.1% | 3.4% ± 0.5% |
| XGB-9 (threshold: default) | 0.0% ± 0.0% | 0.0% ± 0.0% | | 100.0% ± 0.0% | 100.0% ± 0.0% | | 0.0% ± 0.0% | 0.0% ± 0.0% | | 96.2% ± 0.2% | 95.9% ± 0.7% | | 96.4% ± 0.8% | 86.4% ± 2.5% | | 3.0% ± 0.2% | 3.5% ± 0.6% |
| XGB-50 (threshold: ROC) | 95.7% ± 2.0% | 80.0% ± 6.6% | | 81.2% ± 3.4% | 79.9% ± 3.5% | | 17.3% ± 2.4% | 14.3% ± 2.7% | | 99.8% ± 0.1% | 99.0% ± 0.4% | | 96.4% ± 0.7% | 86.8% ± 2.3% | | 3.0% ± 0.1% | 3.4% ± 0.5% |
| XGB-9 (threshold: ROC) | 96.9% ± 1.4% | 81.3% ± 7.0% | | 78.6% ± 3.6% | 77.0% ± 3.5% | | 15.6% ± 2.5% | 13.2% ± 2.3% | | 99.8% ± 0.1% | 99.0% ± 0.4% | | 96.4% ± 0.8% | 86.4% ± 2.5% | | 3.0% ± 0.2% | 3.5% ± 0.6% |
| XGB-50 (threshold: TPR) | 100.0% ± 0.0% | 97.5% ± 2.8% | | 38.4% ± 6.5% | 37.9% ± 6.8% | | 6.2% ± 0.7% | 6.1% ± 1.0% | | 100.0% ± 0.0% | 99.7% ± 0.3% | | 100.0% ± 0.0% | 86.0% ± 2.1% | | 0.7% ± 0.1% | 3.5% ± 0.4% |
| XGB-9 (threshold: ROC) | 100.0% ± 0.0% | 97.6% ± 1.9% | | 38.6% ± 6.2% | 37.5% ± 6.4% | | 6.1% ± 0.7% | 6.3% ± 1.2% | | 100.0% ± 0.0% | 99.8% ± 0.2% | | 100.0% ± 0.0% | 85.6% ± 2.8% | | 0.6% ± 0.2% | 3.6% ± 0.4% |

Supplemental Table 2 | Model performance metrics in dataset 1 training and testing subsets.
AUC: Area under curve; CI: confidence interval; DL: deep learning model; NPV: negative predictive value; PPV: positive predictive value; ROC: receiver operating characteristic; TPR: true positive rate; XGB: eXtreme gradient boosting

|  | Average sensitivity ± 95% CI | | Average specificity ± 95% CI | | Average PPV ± 95% CI | | Average NPV ± 95% CI | | | Average AUC ± 95% CI | | Average Brier Score ± 95% CI | |
| --- | --- | --- | --- | --- | --- | --- | --- | --- | --- | --- | --- | --- | --- |
| **Diagnostic prediction, level 1** | Training | Testing | Training | Testing | Training | Testing | Training | Testing | Training | | Testing | Training | Testing |
| DL-50 (threshold: default) | 92.3% ± 3.4% | 82.3% ± 4.2% | 98.9% ± 0.5% | 97.9% ± 0.8% | 95.6% ± 1.6% | 91.1% ± 2.9% | 98.1% ± 0.8% | 95.6% ± 1.0% | 99.7% ± 0.0% | | 98.5% ± 0.3% | 1.9% ± 0.1% | 3.9% ± 0.4% |
| DL-5 (threshold: default) | 91.7% ± 2.8% | 81.2% ± 5.8% | 99.2% ± 0.4% | 98.2% ± 0.6% | 96.5% ± 1.3% | 91.9% ± 2.8% | 98.0% ± 0.7% | 95.4% ± 1.6% | 99.7% ± 0.0% | | 98.4% ± 0.4% | 1.8% ± 0.1% | 3.9% ± 0.5% |
| DL-50 (threshold: ROC) | 99.4% ± 0.5% | 92.6% ± 2.3% | 95.6% ± 1.3% | 93.6% ± 1.6% | 85.3% ± 3.7% | 78.9% ± 4.0% | 99.8% ± 0.1% | 98.0% ± 0.6% | 99.7% ± 0.0% | | 98.5% ± 0.3% | 1.9% ± 0.1% | 3.9% ± 0.4% |
| DL-5 (threshold: ROC) | 99.3% ± 0.5% | 91.7% ± 1.9% | 95.9% ± 1.4% | 94.0% ± 1.4% | 86.0% ± 4.1% | 79.5% ± 3.3% | 99.8% ± 0.1% | 97.8% ± 0.4% | 99.7% ± 0.0% | | 98.4% ± 0.4% | 1.8% ± 0.1% | 3.9% ± 0.5% |
| DL-50 (threshold: TPR) | 100.0% ± 0.0% | 99.4% ± 0.8% | 78.6% ± 7.1% | 76.9% ± 7.0% | 55.2% ± 7.5% | 53.3% ± 6.3% | 100.0% ± 0.0% | 99.8% ± 0.2% | 99.7% ± 0.0% | | 98.5% ± 0.3% | 1.9% ± 0.1% | 3.9% ± 0.4% |
| DL-5 (threshold: TPR) | 100.0% ± 0.0% | 99.3% ± 1.1% | 81.0% ± 2.3% | 79.3% ± 3.5% | 57.1% ± 3.5% | 54.9% ± 2.6% | 100.0% ± 0.0% | 99.8% ± 0.3% | 99.7% ± 0.0% | | 98.4% ± 0.4% | 1.8% ± 0.1% | 3.9% ± 0.5% |
| XGB-50 (threshold: default) | 98.3% ± 0.8% | 90.1% ± 2.3% | 99.8% ± 0.1% | 98.7% ± 0.4% | 99.3% ± 0.4% | 94.5% ± 1.6% | 99.6% ± 0.2% | 97.5% ± 0.6% | 100.0% ± 0.0% | | 99.5% ± 0.1% | 0.9% ± 0.1% | 2.4% ± 0.3% |
| XGB-19 (threshold: default) | 98.3% ± 1.0% | 89.7% ± 2.6% | 99.8% ± 0.1% | 98.7% ± 0.4% | 99.3% ± 0.5% | 94.8% ± 1.4% | 99.6% ± 0.3% | 97.4% ± 0.7% | 100.0% ± 0.0% | | 99.5% ± 0.1% | 0.9% ± 0.1% | 2.4% ± 0.3% |
| XGB-50 (threshold: ROC) | 99.9% ± 0.1% | 96.6% ± 1.4% | 98.1% ± 0.6% | 96.3% ± 0.9% | 92.9% ± 2.1% | 86.9% ± 2.7% | 100.0% ± 0.0% | 99.1% ± 0.4% | 100.0% ± 0.0% | | 99.5% ± 0.1% | 0.9% ± 0.1% | 2.4% ± 0.3% |
| XGB-19 (threshold: ROC) | 99.9% ± 0.1% | 96.4% ± 1.3% | 98.2% ± 0.5% | 96.5% ± 0.8% | 93.4% ± 1.8% | 87.5% ± 2.6% | 100.0% ± 0.0% | 99.1% ± 0.4% | 100.0% ± 0.0% | | 99.5% ± 0.1% | 0.9% ± 0.1% | 2.4% ± 0.3% |
| XGB-50 (threshold: TPR) | 100.0% ± 0.0% | 99.4% ± 0.6% | 90.0% ± 2.3% | 88.9% ± 2.0% | 71.9% ± 4.5% | 69.8% ± 4.1% | 100.0% ± 0.0% | 99.8% ± 0.2% | 100.0% ± 0.0% | | 99.5% ± 0.1% | 0.9% ± 0.1% | 2.4% ± 0.3% |
| XGB-19 (threshold: TPR) | 100.0% ± 0.0% | 99.6% ± 0.5% | 89.5% ± 2.6% | 88.6% ± 2.1% | 70.9% ± 5.1% | 69.1% ± 3.8% | 100.0% ± 0.0% | 99.9% ± 0.1% | 100.0% ± 0.0% | | 99.5% ± 0.1% | 0.9% ± 0.1% | 2.4% ± 0.3% |
|  |  |  |  |  |  |  |  |  |  | |  |  |  |
|  |  | |  | |  | |  | |  | | |  | |
|  | Average sensitivity ± 95% CI | | Average specificity ± 95% CI | | Average PPV ± 95% CI | | Average NPV ± 95% CI | | Average AUC ± 95% CI | | | Average Brier Score ± 95% CI | |
| **Diagnostic prediction, level 2** | Training | Testing | Training | Testing | Training | Testing | Training | Testing | Training | | Testing | Training | Testing |
| DL-50 (threshold: default) | 89.1% ± 5.8% | 69.1% ± 6.7% | 99.8% ± 0.1% | 99.0% ± 0.4% | 97.5% ± 1.4% | 84.6% ± 5.3% | 99.2% ± 0.4% | 97.7% ± 0.6% | 99.9% ± 0.0% | | 98.0% ± 0.6% | 0.8% ± 0.1% | 2.3% ± 0.3% |
| DL-5 (threshold: default) | 87.2% ± 6.5% | 73.0% ± 9.6% | 99.8% ± 0.1% | 99.2% ± 0.3% | 97.8% ± 1.3% | 87.0% ± 2.7% | 99.1% ± 0.4% | 98.0% ± 0.7% | 99.9% ± 0.0% | | 98.2% ± 0.8% | 0.8% ± 0.1% | 2.1% ± 0.3% |
| DL-50 (threshold: ROC) | 100.0% ± 0.0% | 92.8% ± 4.1% | 94.5% ± 2.1% | 92.8% ± 2.1% | 58.4% ± 9.3% | 50.1% ± 7.1% | 100.0% ± 0.0% | 99.4% ± 0.3% | 99.9% ± 0.0% | | 98.0% ± 0.6% | 0.8% ± 0.1% | 2.3% ± 0.3% |
| DL-5 (threshold: ROC) | 100.0% ± 0.0% | 94.9% ± 3.5% | 93.9% ± 2.4% | 91.7% ± 2.4% | 56.2% ± 8.8% | 47.2% ± 7.9% | 100.0% ± 0.0% | 99.6% ± 0.3% | 99.9% ± 0.0% | | 98.2% ± 0.8% | 0.8% ± 0.1% | 2.1% ± 0.3% |
| DL-50 (threshold: TPR) | 100.0% ± 0.0% | 99.1% ± 1.6% | 73.5% ± 13.6% | 72.5% ± 13.4% | 25.2% ± 9.9% | 24.3% ± 8.7% | 100.0% ± 0.0% | 99.9% ± 0.1% | 99.9% ± 0.0% | | 98.0% ± 0.6% | 0.8% ± 0.1% | 2.3% ± 0.3% |
| DL-5 (threshold: TPR) | 100.0% ± 0.0% | 98.3% ± 2.8% | 74.3% ± 18.2% | 73.1% ± 17.4% | 30.5% ± 16.7% | 27.1% ± 11.4% | 100.0% ± 0.0% | 99.9% ± 0.2% | 99.9% ± 0.0% | | 98.2% ± 0.8% | 0.8% ± 0.1% | 2.1% ± 0.3% |
| XGB-50 (threshold: default) | 86.1% ± 6.7% | 59.6% ± 6.9% | 100.0% ± 0.0% | 99.5% ± 0.3% | 99.9% ± 0.2% | 90.3% ± 4.6% | 99.0% ± 0.5% | 97.0% ± 0.6% | 100.0% ± 0.0% | | 98.3% ± 0.6% | 0.9% ± 0.1% | 2.4% ± 0.4% |
| XGB-19 (threshold: default) | 85.7% ± 7.0% | 59.9% ± 6.8% | 100.0% ± 0.0% | 99.5% ± 0.2% | 99.9% ± 0.2% | 90.6% ± 4.0% | 99.0% ± 0.5% | 97.1% ± 0.6% | 100.0% ± 0.0% | | 98.4% ± 0.5% | 0.9% ± 0.1% | 2.4% ± 0.4% |
| XGB-50 (threshold: ROC) | 99.9% ± 0.1% | 94.3% ± 3.3% | 94.6% ± 1.3% | 93.3% ± 1.5% | 58.3% ± 5.9% | 51.8% ± 5.3% | 100.0% ± 0.0% | 99.5% ± 0.3% | 100.0% ± 0.0% | | 98.3% ± 0.6% | 0.9% ± 0.1% | 2.4% ± 0.4% |
| XGB-19 (threshold: ROC) | 99.9% ± 0.2% | 94.4% ± 3.4% | 94.3% ± 1.4% | 93.0% ± 1.7% | 56.7% ± 6.3% | 51.0% ± 5.4% | 100.0% ± 0.0% | 99.5% ± 0.3% | 100.0% ± 0.0% | | 98.4% ± 0.5% | 0.9% ± 0.1% | 2.4% ± 0.4% |
| XGB-50 (threshold: TPR) | 100.0% ± 0.0% | 98.8% ± 1.2% | 85.0% ± 4.3% | 84.0% ± 4.6% | 33.7% ± 5.7% | 32.6% ± 5.8% | 100.0% ± 0.0% | 99.9% ± 0.1% | 100.0% ± 0.0% | | 98.3% ± 0.6% | 0.9% ± 0.1% | 2.4% ± 0.4% |
| XGB-19 (threshold: TPR) | 100.0% ± 0.0% | 98.9% ± 0.9% | 84.5% ± 4.8% | 83.7% ± 5.4% | 33.2% ± 6.0% | 32.4% ± 6.1% | 100.0% ± 0.0% | 99.9% ± 0.1% | 100.0% ± 0.0% | | 98.4% ± 0.5% | 0.9% ± 0.1% | 2.4% ± 0.4% |
|  |  |  |  |  |  |  |  |  |  | |  |  |  |
|  |  | |  | |  | |  | |  | | |  | |
|  | Average sensitivity ± 95% CI | | Average specificity ± 95% CI | | Average PPV ± 95% CI | | Average NPV ± 95% CI | | Average AUC ± 95% CI | | | Average Brier Score ± 95% CI | |
| **Event prediction** | Training | Testing | Training | Testing | Training | Testing | Training | Testing | Training | | Testing | Training | Testing |
| XGB-50 (threshold: default) | 0.0% ± 0.0% | 0.0% ± 0.0% | 100.0% ± 0.0% | 100.0% ± 0.0% | 0.0% ± 0.0% | 0.0% ± 0.0% | 95.2% ± 0.1% | 95.2% ± 0.5% | 97.3% ± 0.7% | | 83.8% ± 2.1% | 3.7% ± 0.1% | 4.1% ± 0.4% |
| XGB-9 (threshold: default) | 0.0% ± 0.0% | 0.0% ± 0.0% | 100.0% ± 0.0% | 100.0% ± 0.0% | 0.0% ± 0.0% | 0.0% ± 0.0% | 95.2% ± 0.2% | 95.3% ± 0.7% | 97.4% ± 0.9% | | 84.0% ± 2.2% | 3.7% ± 0.1% | 4.1% ± 0.6% |
| XGB-50 (threshold: ROC) | 98.0% ± 0.9% | 81.0% ± 6.9% | 74.2% ± 5.8% | 71.6% ± 5.5% | 16.7% ± 3.1% | 12.7% ± 2.2% | 99.9% ± 0.1% | 98.7% ± 0.5% | 97.3% ± 0.7% | | 83.8% ± 2.1% | 3.7% ± 0.1% | 4.1% ± 0.4% |
| XGB-9 (threshold: ROC) | 98.7% ± 0.8% | 84.8% ± 6.8% | 69.5% ± 6.3% | 67.7% ± 6.0% | 14.6% ± 3.0% | 11.7% ± 2.2% | 99.9% ± 0.1% | 98.9% ± 0.4% | 97.4% ± 0.9% | | 84.0% ± 2.2% | 3.7% ± 0.1% | 4.1% ± 0.6% |
| XGB-50 (threshold: TPR) | 100.0% ± 0.0% | 97.8% ± 1.9% | 20.9% ± 5.9% | 19.9% ± 6.0% | 6.0% ± 0.5% | 5.8% ± 0.7% | 100.0% ± 0.0% | 99.5% ± 0.4% | 100.0% ± 0.0% | | 83.8% ± 2.3% | 0.7% ± 0.2% | 4.0% ± 0.4% |
| XGB-9 (threshold: ROC) | 100.0% ± 0.0% | 97.2% ± 1.8% | 21.0% ± 5.4% | 20.3% ± 5.5% | 6.1% ± 0.5% | 5.7% ± 0.9% | 100.0% ± 0.0% | 99.3% ± 0.4% | 100.0% ± 0.0% | | 83.8% ± 3.2% | 0.7% ± 0.2% | 4.0% ± 0.4% |

Supplemental Table 3 | Model performance metrics in dataset 2 training and testing subsets.
AUC: Area under curve; CI: confidence interval; DL: deep learning model; NPV: negative predictive value; PPV: positive predictive value; ROC: receiver operating characteristic; TPR: true positive rate; XGB: eXtreme gradient boosting

|  |  | 5-class results (DL models) | | | | | | | | | | 3-class results (XGB models) | | | | | |
| --- | --- | --- | --- | --- | --- | --- | --- | --- | --- | --- | --- | --- | --- | --- | --- | --- | --- |
|  |  | Non-elevated | | Chronic myocardial injury | | Acute non-ischaemic myocardial injury | | Type 2 MI | | Type 1 MI | | Non-elevated/chronic myocardial injury | | Type 2 MI/Acute non-ischaemic myocardial injury | | Type 1 MI | |
|  | Model | Training | Testing | Training | Testing | Training | Testing | Training | Testing | Training | Testing | Training | Testing | Training | Testing | Training | Testing |
| Diagnostic prediction – overall datasets | DL-50 | 49.8% | 62.4% | 31.8% | 12.8% | 8.3% | 7.7% | 4.4% | 8.7% | 5.7% | 8.4% |  |  |  |  |  |  |
|  | DL-5 | 49.9% | 62.4% | 31.7% | 12.9% | 8.2% | 7.7% | 4.4% | 8.3% | 5.8% | 8.7% |  |  |  |  |  |  |
|  | XGB-50 |  |  |  |  |  |  |  |  |  |  | 81.6% | 75.2% | 12.7% | 16.4% | 5.7% | 8.4% |
|  | XGB-19 |  |  |  |  |  |  |  |  |  |  | 81.5% | 75.3% | 12.7% | 16.4% | 5.8% | 8.3% |
| Diagnostic prediction – D1  (n=1150) | DL-50 | 61.2% | 61.2% | 21.9% | 21.9% | 8.5% | 8.4% | 3.7% | 3.7% | 4.8% | 4.8% |  |  |  |  |  |  |
|  | DL-5 | 61.1% | 61.0% | 22.0% | 21.8% | 8.3% | 8.8% | 3.8% | 3.6% | 4.8% | 4.7% |  |  |  |  |  |  |
|  | XGB-50 |  |  |  |  |  |  |  |  |  |  | 83.0% | 83.1% | 12.2% | 12.1% | 4.8% | 4.8% |
|  | XGB-19 |  |  |  |  |  |  |  |  |  |  | 83.0% | 83.2% | 12.2% | 12.2% | 4.8% | 4.6% |
| Diagnostic prediction – D2  (n=1050) | DL-50 | 36.8% | 36.6% | 43.0% | 43.0% | 8.1% | 8.3% | 5.2% | 5.1% | 6.8% | 7.0% |  |  |  |  |  |  |
|  | DL-5 | 37.3% | 36.4% | 42.6% | 43.5% | 8.1% | 8.0% | 5.1% | 5.1% | 6.8% | 6.9% |  |  |  |  |  |  |
|  | XGB-50 |  |  |  |  |  |  |  |  |  |  | 79.9% | 79.6% | 13.3% | 13.4% | 6.8% | 7.0% |
|  | XGB-19 |  |  |  |  |  |  |  |  |  |  | 79.9% | 79.7% | 13.3% | 13.3% | 6.8% | 7.0% |

Supplemental Table 4 | Prevalence of each troponin profile for diagnostic prediction models within the training and testing subsets
D1: dataset 1; D2: dataset 2; DL: deep learning; MI: myocardial infarction; XGB: eXtreme gradient boosting

|  |  | 30-day death or MI | | | |
| --- | --- | --- | --- | --- | --- |
|  |  | No | | Yes | |
|  | Model | Training | Testing | Training | Testing |
| Event prediction – overall | XGB-50 | 95.7% | 94.9% | 4.3% | 5.1% |
|  | XGB-9 | 95.7% | 94.9% | 4.3% | 5.1% |
| Event prediction – D1 | XGB-50 | 96.1% | 96.0% | 3.9% | 4.0% |
|  | XGB-9 | 96.2% | 95.9% | 3.8% | 4.1% |
| Event prediction – D1 | XGB-50 | 95.2% | 95.2% | 4.8% | 4.6% |
|  | XGB-9 | 95.2% | 95.3% | 4.8% | 4.7% |

Supplemental Table 5 | Prevalence of 30-day death or MI outcomes for event prediction models within the training and testing subsets
D1: dataset 1; D2: dataset 2; DL: deep learning; MI: myocardial infarction; XGB: eXtreme gradient boosting

| Features | Dataset 1  (n=5958) | Dataset 2  (n=5248) | Dataset 3  (n=6627) |
| --- | --- | --- | --- |
| Age | 100% | 100% | 100% |
| Gender | 100% | 100% | 100% |
| Prior co-morbidities |  |  |  |
| Hypertension | 100% | 100% | 100% |
| Diabetes | 100% | 100% | 100% |
| Smoking | 100% | 100% | 100% |
| AMI | 100% | 100% | 100% |
| Heart failure | 100% | 100% | 100% |
| CVA | 100% | 100% | 100% |
| COPD | 100% | 100% | 100% |
| CKD | 100% | 100% | 100% |
| eGFR | 99.2% | 99.9% | 99.2% |
| Troponin data^a^ | 100% | 100% | 100% |
| ECG data^b^ | 0% | 100% | 0% |
| Pathology data |  |  |  |
| Serum Albumin | 92.5% | 97.5% | 95.4% |
| NT-pro BNP | 2.1% | 26.9% | 15.2% |
| Serum Creatinine | 97.2% | 99.9% | 99.1% |
| C-reactive protein | 51.6% | 92.0% | 82.1% |
| D-dimer | 12.7% | 21.3% | 15.3% |
| Serum ferritin | 3.5% | 16.6% | 13.1% |
| Fibrin | 1.1% | 2.6% | 3.5% |
| Haemoglobin | 96.9% | 99.8% | 99.1% |
| HbA1c | 11.3% | 21.5% | 16.4% |
| Platelet count | 96.3% | 99.7% | 99.0% |
| Mean platelet volume | 31.5% | 99.2% | 98.7% |
| TSH | 12.8% | 27.4% | 21.2% |
| Serum Urate | 42.3% | 47.8% | 50.2% |
| Serum Urea | 97.2% | 99.9% | 99.2% |
| WBC | 96.9% | 99.8% | 99.1% |

Supplemental Table 6 | Data availability of all features
ACS: Acute Coronary Syndrome; AMI: acute myocardial infarction; BNP: brain natriuretic peptide; COPD: chronic obstructive pulmonary disease; CKD: chronic kidney disease; CVA: cerebrovascular accident; eGFR: estimated glomerular filtration rate; TSH: thyroid stimulating hormone; WBC: white blood cell count
^a^Troponin data included 10 variables as outlined in the Supplemental methods (Data pre-processing and transformation: feature engineering/extraction/selection)
^b^ECG data included the 12 binary variables as listed in Supplemental Table 5.

Supplemental Table 7 | Availability of ECG variables from Dataset 2

| ECG data, % | Dataset 2 |
| --- | --- |
| Sinus rhythm | 56.4% |
| Left ventricular hypertrophy | 11.6% |
| Right bundle branch block | 12.6% |
| Left bundle branch block | 6.3% |
| T wave inversion | 30.0% |
| ST depression | 21.3% |
| Acute injury | 9.7% |
| Old injury | 24.5% |
| AV block | 10.2% |
| Paced rhythm | 6.0% |
| Ventricular tachycardia | 0.7% |
| Atrial fibrillation | 16.4% |

AV: atrioventricular; ECG: electrocardiogram

Supplemental Table 8 | Model performance with varying input data

|  | Diagnostic: DL-50 | | | | Diagnostic: DL-5 | | | | Diagnostic: XGB-50 | | | | Diagnostic: XGB-19 | | | | Event: XGB-50 | | Event: XGB-9 | |  |
| --- | --- | --- | --- | --- | --- | --- | --- | --- | --- | --- | --- | --- | --- | --- | --- | --- | --- | --- | --- | --- | --- |
|  | Level 1 | | Level 2 | | Level 1 | | Level 2 | | Level 1 | | Level 2 | | Level 1 | | Level 2 | |  | |  | |  |
|  | AUC | 95%CI^a^ | AUC | 95%CI^a^ | AUC | 95%CI^a^ | AUC | 95%CI^a^ | AUC | 95%CI^a^ | AUC | 95%CI^a^ | AUC | 95%CI^a^ | AUC | 95%CI^a^ | AUC | 95%CI^a^ | AUC | 95%CI^a^ | |
| Clinical data only | 0.716 | 0.009 | 0.636 | 0.008 | 0.716 | 0.004 | 0.637 | 0.004 | 0.699 | 0.009 | 0.651 | 0.006 | 0.7 | 0.008 | 0.651 | 0.005 | 0.76 | 0.006 | 0.764 | 0.004 | |
| Troponin data only | 0.991 | 0.002 | 0.924 | 0.006 | 0.991 | 0.001 | 0.922 | 0.008 | 0.992 | 0.001 | 0.922 | 0.005 | 0.992 | 0.001 | 0.923 | 0.003 | 0.837 | 0.008 | 0.837 | 0.005 | |
| Clinical data + troponin data | 0.989 | 0.002 | 0.931 | 0.008 | 0.988 | 0.001 | 0.924 | 0.014 | 0.992 | 0.001 | 0.933 | 0.003 | 0.992 | 0.002 | 0.932 | 0.004 | 0.849 | 0.007 | 0.852 | 0.004 | |
| Clinical data + troponin data + ECG | 0.988 | 0.002 | 0.931 | 0.008 | 0.987 | 0.002 | 0.925 | 0.015 | 0.992 | 0.001 | 0.935 | 0.002 | 0.992 | 0.001 | 0.935 | 0.003 | 0.85 | 0.006 | 0.849 | 0.005 | |
| Clinical data + troponin data + ECG + renal function + haemoglobin + WBC | 0.988 | 0.002 | 0.937 | 0.01 | 0.987 | 0.002 | 0.934 | 0.015 | 0.992 | 0.001 | 0.943 | 0.002 | 0.992 | 0.001 | 0.942 | 0.003 | 0.866 | 0.006 | 0.866 | 0.007 | |
| All input data | 0.988 | 0.002 | 0.946 | 0.009 | 0.988 | 0.001 | 0.938 | 0.019 | 0.992 | 0.001 | 0.955 | 0.002 | 0.992 | 0.001 | 0.955 | 0.003 | 0.885 | 0.005 | 0.884 | 0.008 | |

*AUC: Area under curve; CI: confidence interval; DL: deep learning; ECG: electrocardiogram; WBC: white blood cell count; XGB: eXtreme gradient boosting*

*^a^95% confidence interval (half-length)*

Supplemental Table 9 | Model performance in age sub-groups

|  | Diagnostic prediction | | | | | | | | | | | | | | | | Event prediction | | | |
| --- | --- | --- | --- | --- | --- | --- | --- | --- | --- | --- | --- | --- | --- | --- | --- | --- | --- | --- | --- | --- |
|  | DL-50 | | | | DL-5 | | | | XGB-50 | | | | XGB-19 | | | | XGB-50 | | XGB-9 | |
|  | Level 1 | | Level 2 | | Level 1 | | Level 2 | | Level 1 | | Level 2 | | Level 1 | | Level 2 | |  |  |  |  |
| Age | AUC | 95%CI^a^ | AUC | 95%CI^a^ | AUC | 95%CI^a^ | AUC | 95%CI^a^ | AUC | 95%CI^a^ | AUC | 95%CI^a^ | AUC | 95%CI^a^ | AUC | 95%CI^a^ | AUC | 95%CI^a^ | AUC | 95%CI^a^ |
| <50 | 0.991 | 0.002 | 0.98 | 0.009 | 0.991 | 0 | 0.972 | 0.017 | 0.995 | 0.002 | 0.99 | 0.002 | 0.991 | 0.001 | 0.989 | 0.002 | 0.912 | 0.023 | 0.912 | 0.031 |
| 50-60 | 0.986 | 0.004 | 0.968 | 0.008 | 0.987 | 0.002 | 0.961 | 0.016 | 0.989 | 0.001 | 0.974 | 0.002 | 0.989 | 0.001 | 0.974 | 0.002 | 0.922 | 0.023 | 0.922 | 0.024 |
| 60-70 | 0.989 | 0.002 | 0.945 | 0.009 | 0.989 | 0.001 | 0.936 | 0.018 | 0.992 | 0.001 | 0.954 | 0.003 | 0.992 | 0.001 | 0.954 | 0.003 | 0.87 | 0.015 | 0.868 | 0.015 |
| 70-80 | 0.981 | 0.003 | 0.912 | 0.011 | 0.981 | 0.001 | 0.91 | 0.019 | 0.99 | 0.002 | 0.928 | 0.005 | 0.99 | 0.001 | 0.928 | 0.005 | 0.822 | 0.01 | 0.82 | 0.009 |
| >80 | 0.973 | 0.002 | 0.893 | 0.009 | 0.974 | 0.001 | 0.887 | 0.018 | 0.983 | 0.002 | 0.897 | 0.006 | 0.984 | 0.002 | 0.897 | 0.007 | 0.763 | 0.012 | 0.758 | 0.018 |

*AUC: Area under curve; CI: confidence interval; DL: deep learning; XGB: eXtreme gradient boosting*

*^a^95% confidence interval (half-length)*

Supplemental Table 10 | Model performance between genders

|  |  | Male | | Female | |
| --- | --- | --- | --- | --- | --- |
|  |  | AUC | 95%CI^a^ | AUC | 95%CI^a^ |
| Diagnostic | DL-50: Level 1 | 0.99 | 0.002 | 0.984 | 0.002 |
|  | DL-50: Level 2 | 0.945 | 0.013 | 0.941 | 0.007 |
|  | DL-5: Level 1 | 0.99 | 0.001 | 0.985 | 0.001 |
|  | DL-5: Level 2 | 0.935 | 0.027 | 0.935 | 0.015 |
|  | XGB-50: Level 1 | 0.994 | 0.001 | 0.99 | 0.001 |
|  | XGB-50: Level 2 | 0.96 | 0.002 | 0.948 | 0.003 |
|  | XGB-19: Level 1 | 0.994 | 0.001 | 0.99 | 0.001 |
|  | XGB-19: Level 2 | 0.959 | 0.003 | 0.948 | 0.003 |
| Event | XGB-9 | 0.914 | 0.011 | 0.854 | 0.006 |
|  | XGB-50 | 0.914 | 0.006 | 0.856 | 0.006 |

*AUC: Area under curve; CI: confidence interval; DL: deep learning; XGB: eXtreme gradient boosting*

*^a^95% confidence interval (half-length)*

Supplemental Table 11 | Model performance with varying time of troponin data

|  | Diagnostic prediction | | | | | | | | | | | | Event prediction | | | | | | | |
| --- | --- | --- | --- | --- | --- | --- | --- | --- | --- | --- | --- | --- | --- | --- | --- | --- | --- | --- | --- | --- |
| DL-50 | | | | | DL-5 | | | | XGB-50 | | | | XGB-19 | | | | Event: XGB-50 | | Event: XGB-9 | |
|  | Level 1 | | Level 2 | | Level 1 | | Level 2 | | Level 1 | | Level 2 | | Level 1 | | Level 2 | |  | |  | |
| Troponin results | AUC | 95%CI^a^ | AUC | 95%CI^a^ | AUC | 95%CI^a^ | AUC | 95%CI^a^ | AUC | 95%CI^a^ | AUC | 95%CI^a^ | AUC | 95%CI^a^ | AUC | 95%CI^a^ | AUC | 95%CI^a^ | AUC | 95%CI^a^ |
| <3 hours | 0.931 | 0.013 | 0.874 | 0.025 | 0.934 | 0.001 | 0.856 | 0.045 | 0.943 | 0.006 | 0.884 | 0.006 | 0.943 | 0.003 | 0.884 | 0.006 | 0.895 | 0.006 | 0.893 | 0.009 |
| <6 hours | 0.963 | 0.008 | 0.916 | 0.016 | 0.965 | 0.005 | 0.904 | 0.031 | 0.972 | 0.003 | 0.928 | 0.003 | 0.972 | 0.002 | 0.928 | 0.004 | 0.9 | 0.006 | 0.897 | 0.01 |
| <12 hours | 0.978 | 0.004 | 0.936 | 0.012 | 0.979 | 0.002 | 0.926 | 0.024 | 0.985 | 0.002 | 0.948 | 0.002 | 0.985 | 0.001 | 0.947 | 0.003 | 0.901 | 0.006 | 0.9 | 0.01 |
| <24 hours | 0.988 | 0.002 | 0.946 | 0.009 | 0.988 | 0.001 | 0.938 | 0.019 | 0.992 | 0.001 | 0.955 | 0.002 | 0.992 | 0.001 | 0.955 | 0.003 | 0.885 | 0.005 | 0.884 | 0.008 |

*AUC: Area under curve; CI: confidence interval; DL: deep learning; XGB: eXtreme gradient boosting*

*^a^95% confidence interval (half-length)*

Supplemental Table 12 | Calibration statistics

|  |  | Calibration slope ± 95% CI | Calibration intercept ± 95% CI | Mean calibration ± 95% CI |
| --- | --- | --- | --- | --- |
| Diagnostic | DL-50: Level 1 | 1.0 ± 0.3 | 1.8 ± 1.9 | 2.9% ± 0.3% |
|  | DL-50: Level 2 | 0.7 ± 0.4 | 0.5 ± 2.2 | 2.1% ± 0.4% |
|  | DL-5: Level 1 | 1.0 ± 0.1 | 1.7 ± 1.8 | 2.9% ± 0.2% |
|  | DL-5: Level 2 | 0.5 ± 0.4 | 0.9 ± 2.6 | 2.2% ± 0.3% |
|  | XGB-50: Level 1 | 1.6 ± 0.5 | 2.0 ± 1.2 | 3.3% ± 0.2% |
|  | XGB-50: Level 2 | 1.3 ± 0.7 | 0.9 ± 1.5 | 2.1% ± 0.2% |
|  | XGB-19: Level 1 | 1.8 ± 0.6 | 2.6 ± 1.2 | 3.4% ± 0.3% |
|  | XGB-19: Level 2 | 1.3 ± 0.7 | 0.6 ± 1.5 | 2.1% ± 0.2% |
| Event | XGB-50 | 2.1 ± 2.1 | 3.9 ± 7.4 | 1.5% ± 0.3% |
|  | XGB-9 | 1.7 ± 1.4 | 2.7 ± 4.1 | 1.7% ± 0.3% |

*AUC: Area under curve; CI: confidence interval; DL: deep learning; XGB: eXtreme gradient boosting*

*Supplemental Figure 1 |* *Model performance and feature importance in lower repetition models. Panel A-E: ROC curves for diagnostic and event prediction models. Panel F-I: Feature importance. Panel J: Model performance according to input data.*

*AUC: Area under curve; D1: dataset 1; D2: dataset 2; DL: deep learning; ECG: electrocardiogram; eGFR: estimated glomerular filtration rate; WBC: white blood cell count; XGB: eXtreme gradient boosting*

Supplemental Figure 2 | Model performance in subgroups. Panel A-B: Age; C-D: Varying time of troponin data; E-F: Gender
AUC: Area under curve; DL: deep learning; XGB: eXtreme gradient boosting


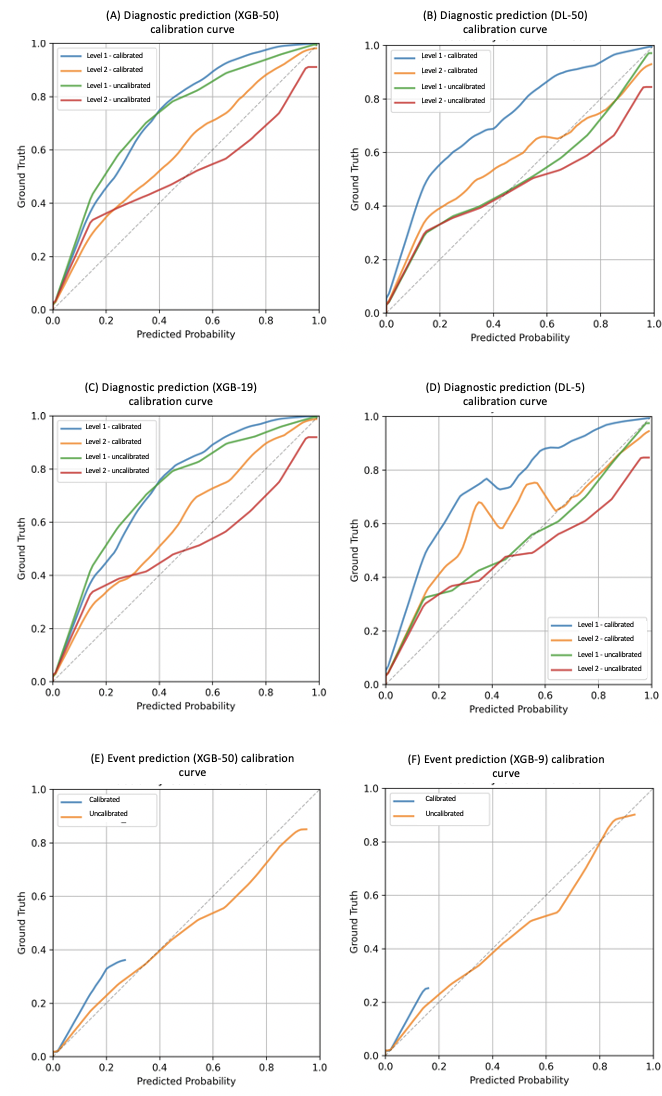


*Supplemental Figure 3 | Calibration curves. DL: deep learning; XGB: eXtreme gradient boosting*

**

*Supplemental Figure 4 | Process of dataset splitting and model development
DL: deep learning; XGB: eXtreme gradient boosting*

**Supplemental Reference:**

1. Astley CM, Beltrame JF, Zeitz C et al. Study design of embracing high-sensitivity troponin effectively: The value of more information: A randomized comparison. Contemporary Clinical Trials 2014;39:183-190.

2. Papendick C, Blyth A, Seshadri A et al. A randomized trial of a 1-hour troponin T protocol in suspected acute coronary syndromes: Design of the Rapid Assessment of Possible ACS In the emergency Department with high sensitivity Troponin T (RAPID-TnT) study. American Heart Journal 2017;190:25-33.

3. Sergey I, Szegedy C. Batch Normalization: Accelerating Deep Network Training by Reducing Internal Covariate Shift. International conference on machine learning PMLR 2015:448-456.

4. Nair V, Hinton GE. Rectified Linear Units Improve Restricted Boltzmann Machines. Icml'10 2010:807–814.

5. Srivastava N, Hinton G, Krizhevsky A, Sutskever I, Salakhutdinov R. Dropout: A Simple Way to Prevent Neural Networks from Overfitting. Journal of Machine Learning Research 2014;15:1929-1958.

6. Paszke A, Gross S, Massa F et al. PyTorch: An Imperative Style, High-Performance Deep Learning Library. ArXiv 2019;abs/1912.01703.
